# A short upstream promoter region mediates transcriptional regulation of the mouse *doublecortin *gene in differentiating neurons

**DOI:** 10.1186/1471-2202-11-64

**Published:** 2010-05-28

**Authors:** Marie Piens, Marc Muller, Morgan Bodson, Gregory Baudouin, Jean-Christophe Plumier

**Affiliations:** 1 Laboratory for Animal Physiology, Université de Liège, B-4000 Liège, Sart-Tilman, Belgium; 2 Laboratory for Molecular Biology and Genetic Engineering - GIGA-R, Université de Liège, B-4000 Liège, Sart-Tilman, Belgium; 3 GIGA-Neurosciences, Université de Liège, B-4000 Liège, Sart-Tilman, Belgium

## Abstract

**Background:**

Doublecortin (Dcx), a MAP (Microtubule-Associated Protein), is transiently expressed in migrating and differentiating neurons and thereby characterizes neuronal precursors and neurogenesis in developing and adult neurogenesis. In addition, reduced *Dcx *expression during development has been related to appearance of brain pathologies. Here, we attempt to unveil the molecular mechanisms controlling *Dcx *gene expression by studying its transcriptional regulation during neuronal differentiation.

**Results:**

To determine and analyze important regulatory sequences of the *Dcx *promoter, we studied a putative regulatory region upstream from the mouse *Dcx *coding region (*pdcx*2kb) and several deletions thereof. These different fragments were used *in vitro *and *in vivo *to drive reporter gene expression. We demonstrated, using transient expression experiments, that *pdcx*2kb is sufficient to control specific reporter gene expression in cerebellar cells and in the developing brain (E14.5). We determined the temporal profile of *Dcx *promoter activity during neuronal differentiation of mouse embryonic stem cells (mESC) and found that transcriptional activation of the *Dcx *gene varies along with neuronal differentiation of mESC. Deletion experiments and sequence comparison of *Dcx *promoters across rodents, human and chicken revealed the importance of a highly conserved sequence in the proximal region of the promoter required for specific and strong expression in neuronal precursors and young neuronal cells. Further analyses revealed the presence in this short sequence of several conserved, putative transcription factor binding sites: LEF/TCF (Lymphoid Enhancer Factor/T-Cell Factor) which are effectors of the canonical Wnt pathway; HNF6/OC2 (Hepatocyte Nuclear Factor-6/Oncecut-2) members of the ONECUT family and NF-Y/CAAT (Nuclear Factor-Y).

**Conclusions:**

Studies of *Dcx *gene regulatory sequences using native, deleted and mutated constructs suggest that fragments located upstream of the *Dcx *coding sequence are sufficient to induce specific Dcx expression *in vitro*: in heterogeneous differentiated neurons from mESC, in primary mouse cerebellar neurons (PND3) and in organotypic slice cultures. Furthermore, a region in the 3'-end region of the *Dcx *promoter is highly conserved across several species and exerts positive control on *Dcx *transcriptional activation. Together, these results indicate that the proximal 3'-end region of the mouse *Dcx *regulatory sequence is essential for *Dcx *gene expression during differentiation of neuronal precursors.

## Background

The *DCX *gene is located on the X chromosome (Xq22.3-q23) and encodes a 40 kDa phosphoprotein of 360 amino acids. The DCX protein is a microtubule-associated protein (MAP) that interacts with and stabilizes the microtubule cytoskeleton [1,2]. This gene is specifically and transiently expressed in proliferating neuronal progenitors and in post-mitotic neuronal precursors during embryonic development and in neurogenic regions of the adult brain [2-5]. *DCX *expression occurs during corticogenesis and is absent during regenerative axonal growth, suggesting that DCX is a selective marker of cells committed to the neuronal lineage in both developing and adult brain [6,7]. One or more mutations in the *DCX *gene cause X-Linked Subcortical Laminar Band Heterotopia (X-SCLH)/Lissencephaly (LIS) [2,8]. This developmental brain malformation syndrome is caused by abnormal neuronal migration leading to a profound cerebral cortical layer disorganization resulting in mental retardation and epilepsy.

Such alterations in cortical lamination observed in humans were not detected in mice with *Dcx *gene deletion; lamination defects were only observed in the hippocampus [9]. In contrast, RNA interference (RNAi)-mediated knock-down of *Dcx *in rodents caused impairment in radial migration of cortical neurons [10,11]. Similarly, mice with mutations of both *Dcx *and *Dclk *(*Doublecortin-like kinase *gene, homolog of the *Dcx *gene) genes presented both disorganized neocortical lamination and severe cytoarchitectural defects of the hippocampus, suggesting redundant functions of *Dcx *and *Dclk *during neuronal migration [12-14].

Neuronal differentiation is a tightly orchestrated time- and location-dependent process in which many extracellular and intrinsic factors are involved [15-17]. Whereas the temporal and spatial *Dcx *expression patterns and Dcx post-translational regulations are well known, *Dcx *transcriptional regulation is poorly understood. However, to understand the mechanisms involved in neuronal differentiation during embryogenesis and more precisely in stages before neuronal determination, it is crucial to investigate the transcriptional gene control in action during neuronal differentiation. In view of the relevance of Dcx as a marker for neurogenesis and considering the importance of understanding *Dcx *gene regulation, we analyzed a putative regulatory region upstream of the mouse *Dcx *gene (*pdcx*2kb) and used it to drive reporter gene expression. We characterized the *Dcx *promoter activity at different time-points during neuronal differentiation of mESC (mouse embryonic stem cells) and we defined a small region as an element required to provide specific and strong expression in neuronal precursors and young neuronal cells.

## Results

**Figure 1:**
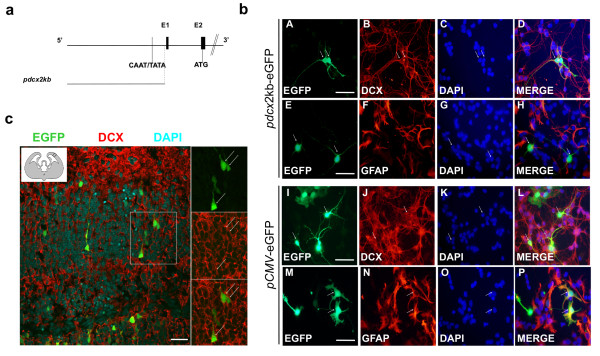
**Neuronal lineage specificity of the doublecortin regulatory sequence (*pdcx*2kb-eGFP)**. **(a) **Schematic representation of the mouse *Dcx *promoter construct for reporter gene expression: black boxes (E1 and E2) represent the first two exons of the *Dcx *gene: the localization of putative CAAT/TATA boxes and ATG start codon are shown **(b) **Cerebellar Granule neurons (CGN) extracted from PND3 mice were transiently transfected with *pdcx*2kb-eGFP (A-H) or *pCMV*-eGFP (I-P). After 72 hrs, expression of eGFP was analyzed using an inverted fluorescence microscope and compared with the neuronal cell-specific marker Dcx or astroglial cell-specific marker GFAP; DAPI was used as a nuclear counterstain. In cells transfected with the *pdcx*2kb plasmid, co-expression was observed between eGFP and Dcx (A-D). No expression overlap is observed between eGFP and GFAP (E-H). Cells transfected with *pCMV*-eGFP plasmid (I-P) present a strong eGFP expression with Dcx (I-L) and with GFAP (M-P). Scale bar equals 50 μm. **(c) **E15 embryonic mouse brains were electroporated with *pdcx*2kb-EGFP plasmid. After 4 days, organotypic slices were sectioned, immunostained with Dcx and counterstained with DAPI. Slices were analyzed by confocal microscopy. The insert shows the location of the microphotograph: eGFP, Dcx and their co-localization are presented. Scale bar equals 50 μm.

To study transcriptional regulation of the mouse *doublecortin *gene (Accession number NT_039718), we selected a 2kb-long fragment upstream of the transcription initiation site of the longest reported *Dcx *mRNA transcript (Accession number NM_001110222). This 2kb-long fragment (*pdcx*2kb) contained TATA and CAAT boxes and was cloned into promoterless reporter (eGFP or Luciferase) vectors for analysis (Figure 1a).

### Cell type-specific activity of the 2kb-long Dcx promoter fragment

We first wanted to determine whether *pdcx*2kb was sufficient to induce transcriptional activity and to drive it specifically in cells expressing the endogenous *Dcx *gene, namely neuronal precursor cells. Two mouse cell types were used: cerebellar neurons from post-natal day 3 mice (PND3) in which endogenous Dcx expression has already been reported [18] and mouse embryonic stem (ESR1) cells.

Cell specificity of *pdcx*2kb transcriptional activity was observed in both heterogeneous cell cultures by transient expression of the eGFP protein upon transfection with *pdcx*2kb-eGFP constructs (Figure 1b). In primary cerebellar cells, eGFP under the control of *pdcx*2kb was detected only in cells expressing the endogenous *Dcx *gene and not in other cell types, such as GFAP-expressing astrocytes (Figure 1b: A-H). Control experiments using the widely active *pCMV*-eGFP construct revealed that eGFP fluorescence was detected indifferently in all cell types, neurons and glial cells alike. Both GFAP- and Dcx-positive cells were fluorescent (Figure 1b: I-P), showing that the transfection process was not cell-specific. Similarly, eGFP expression was only detected in ESR1 cells after neuronal differentiation and was limited to Dcx-positive or βIII-Tubulin-positive cells, confirming *pdcx*2kb selectivity to Dcx-positive neuronal precursor cells *in vitro *(data not shown).

*Ex vivo *electroporation of embryonic mouse brains was also conducted to confirm cell selectivity of *pdcx*2kb activity in a more physiological setting. *pdcx*2kb-eGFP was transfected in brains of E14.5-E15.5 mice, a specific time point of corticogenesis [19]. Dcx expression was observed in migrating cortical neurons originating from progenitor cells located near the ventricular proliferative zone. Following *pdcx*2kb-eGFP transfection, fluorescence was observed around the electroporation site in the proliferative zone of the cerebral cortex. Fluorescent cells displayed morphology similar to that of migrating neuronal precursors (Figure 1c). Moreover, all fluorescent cells also expressed Dcx. Taken together, these qualitative analyses show that the 2kb-long fragment upstream of the *Dcx *gene possesses regulatory sequences sufficient to mimic the activity of the endogenous *Dcx *gene in mouse embryos and to limit its activity to Dcx-positive neuronal precursors.

### Transcriptional activity of the Dcx promoter in differentiating ES cells

To study more efficiently the transcriptional regulation of the mouse *Dcx *gene, we chose to use mouse embryonic stem (ESR1) cells: these cells are able to proliferate indefinitely *in vitro *while retaining pluripotency, and also to differentiate into a large variety of cell types *in vitro*. Hence, mouse ES cells can differentiate into neural precursors able to generate functional neurons [20], astrocytes and oligodendrocytes [21,22]. Using our protocol (see Material and Methods), induction of neuronal differentiation leads to a progressive change in ESR1 cell morphology to a characteristic cobblestone structure and, by neuronal differentiation day 8 (DD8), ESR1 cells begin to form long cellular processes. From DD8/10 on, bipolar cells proliferate and form rosettes.

**Figure 2:**
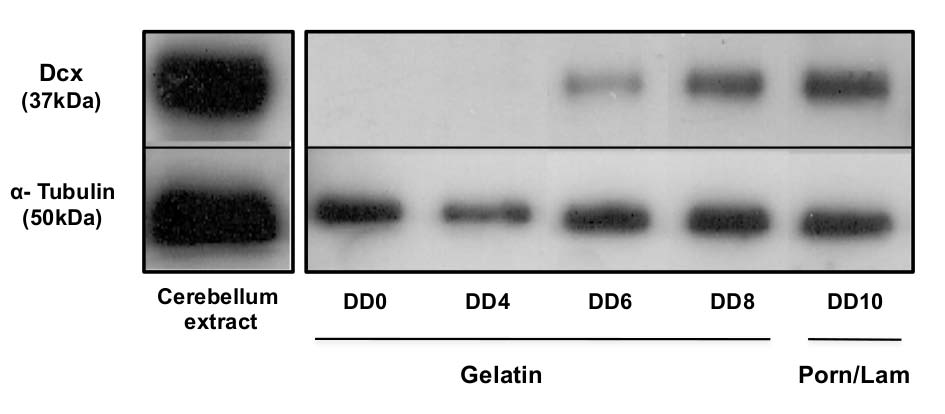
**Confirmation of Dcx expression in cerebellum extracts and embryonic stem cells during neuronal differentiation**. ESR1 cells were subjected to detergent extraction as described in Materials and Methods at day of differentiation 0 (DD0), DD4, DD6, DD8 (on gelatin) and DD10 (on poly-ornithine/laminin). 20 μg of total protein were separated on 10% SDS-PAGE gels, immunoblotted and reacted with the corresponding antibodies; (upper panel) anti-Doublecortin and (lower panel) anti-α-Tubulin.

Dcx protein was detected at DD6 of ESR1 differentiation (Figure 2) and was still present in immature neuronal cells-derived from ESR1 cells at DD8 and DD18. Based on these observations and on the detection of *Dcx *mRNA at DD4, we decided to use ESR1 at DD6 for further analysis of the transcriptional activity of the mouse *Dcx *promoter.

To characterize relevant regions of *pdcx*, we dissected the 2kb-long fragment into shorter fragments of 1.2 kb, 1 kb (obtained by PCR) and 249 bp (obtained by enzymatic restriction), and inserted them upstream of the luciferase reporter gene.

**Figure 3:**
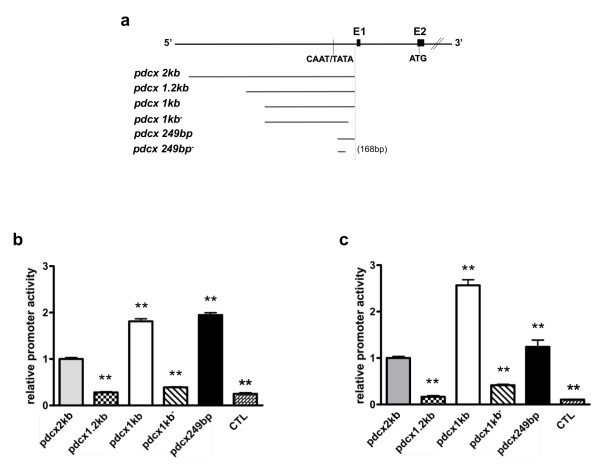
**Transcriptional activity of the mouse *Dcx *upstream regulatory sequences in embryonic stem cells during neuronal differentiation and in cerebellar cells**. **(a) **Schematic representation of the mouse *Dcx *promoter constructs for reporter gene expression. Cells were co-transfected with *Dcx *promoter constructs or the basic vector (promoterless luciferase vector; CTL) and control pSV-β-Galactosidase vector. Luciferase activity (expressed in RLU), of each transfection was determined after 48 hours and normalized to the corresponding internal control, β-Galactosidase activity (OD at 420 nm). **(b) **ESR1 cells were transfected at DD6 program and **(c) **CGN were extracted from PND3 mice and transfected the same day. Each value represents the mean ± SEM of at least three independent transfection experiments, each performed in triplicate. Asterisks mean significantly different from *pdcx*2kb values at *P *< 0.05 (*) or *P *< 0.01 (**).

Comparison of the respective activities of all constructs (Figure 3a) in ESR1 cells at DD8 revealed a higher activity in cells transfected with *pdcx*2kb than in control cells (figure 3b). Transcriptional activities were also higher in cells transfected with shorter fragments (*pdcx*1kb and *pdcx*249bp) than with *pdcx*2kb (Figure 3b). In contrast, the 1.2kb-long *pdcx *fragment did not induce a transcriptional activity different from control, suggesting the presence of a transcriptional repressor region in this 200 bp fragment. In addition, deletion of 79 bp at the 3'end of *pdcx*1kb (*pdcx*1kb-) prevented the *pdcx*1kb-induced increase of activity. This last result suggested the presence of important regulatory domains at the 3'end of *pdcx*, especially in a 79bp-long fragment. Similar effects on the transcriptional activities of the different constructs were observed in PND3 cerebellar cells (Figure 3c), confirming the presence of similar regulatory control mechanisms in neuronal precursors from both origins (ESR1 cells and primary cerebellar cells). However, a discrepancy is observed between the relative activity of *pdcx*1kb and *pdcx*249bp between both cell cultures (Figure 3b and 3c), maybe reflecting the fact that the target regulatory elements necessary for the *Dcx *gene expression are different in two cell types. Indeed, it is quite possible that activators or inhibitor elements, located in *pdcx*1kb and *pdcx*249bp respectively, are most needed during the differentiation of CGN compared to neuroblasts from ESC. At that stage (DD6), ESR1 cells are plated on gelatin-coated plates while cerebellar cells are plated on poly-ornithine substrate (necessary for cell adhesion) (see also below).

**Figure 4:**
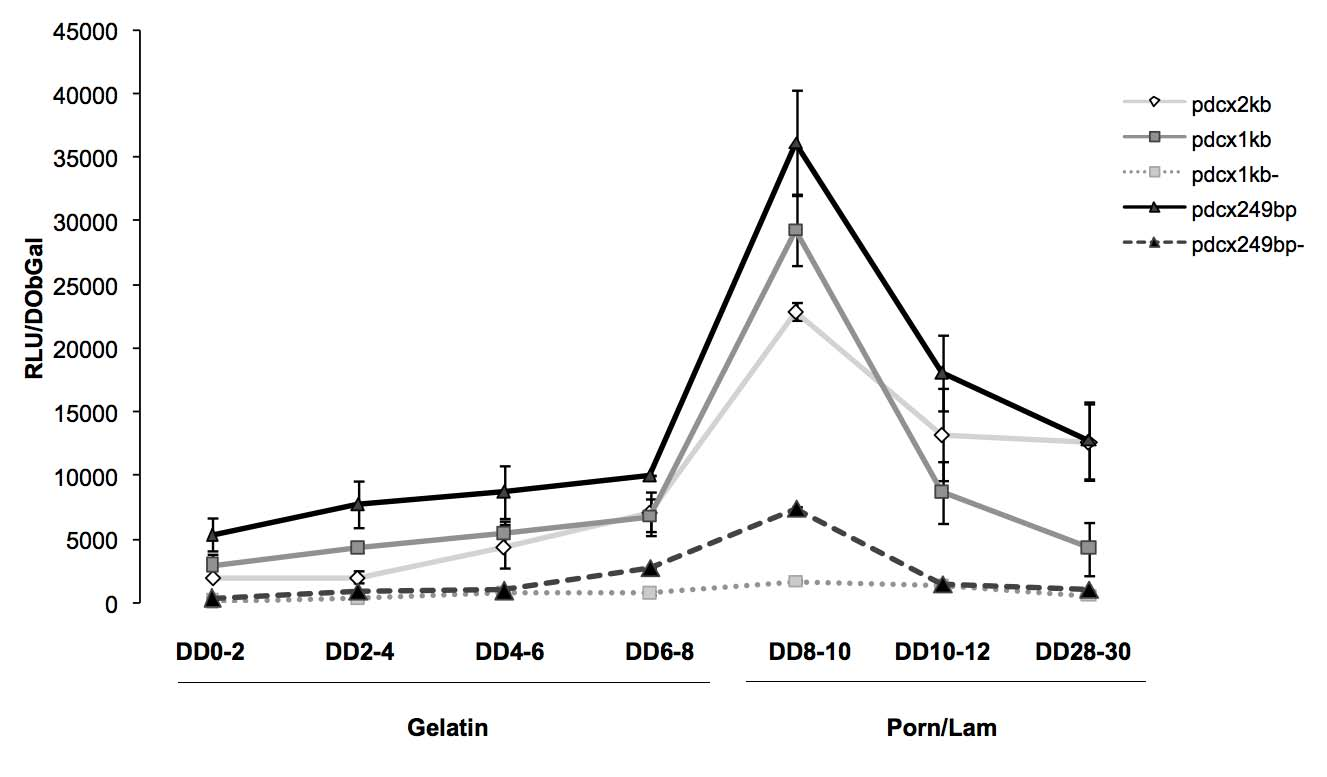
**Transcriptional activity of the mouse *Dcx *upstream regulatory sequences in embryonic stem cells during differentiation**. Every second day, cells were co-transfected with the indicated *Dcx *promoter construct or the promoterless control (CTL). Normalized luciferase activity corresponds to the luciferase activity (expressed in RLU) of each transfection (determined 48 hours later) normalized to the corresponding internal control, β-Galactosidase activity (expressed in OD at 420 nm) (see materials and methods). Each value represents mean ± SEM of at least three independent transfection experiments, each performed in triplicate.

The same relative activities of the promoter fragments were maintained throughout the entire differentiation program in ESR1 cells (Figure 4). Indeed, when ESR1 cells were transfected every second day throughout the entire differentiation period and the luciferase activity measured 48 hours later, increasing levels of activities were observed along the process with a peak at the transfer onto poly-ornithine/lamin substrate for constructs *pdcx*2kb, *pdcx*1kb and *pdcx*249bp. The time-course studies confirmed the inhibition or the significant reduction of the transcriptional activity of *pdcx *at all stages by deletion of 79b at the 3' end of the *pdcx *sequences in constructs *pdcx*1kb and *pdcx*249bp (*pdcx*1kb^- ^and *pdcx*249bp^-^, respectively). Finally, we also examined the cellular specificity of *pdcx*1kb and *pdcx*249bp in differentiated ESR1 and PND3 cerebellar cells and confirmed that, similar to the *pdcx*2kb construct (Figure 1b), both *pdcx*1kb and *pdcx*249bp fragments restricted the expression of the eGFP reporter gene in Dcx-expressing cells (data not shown).

### Transcription factors regulating the mouse Dcx promoter

**Figure 5:**
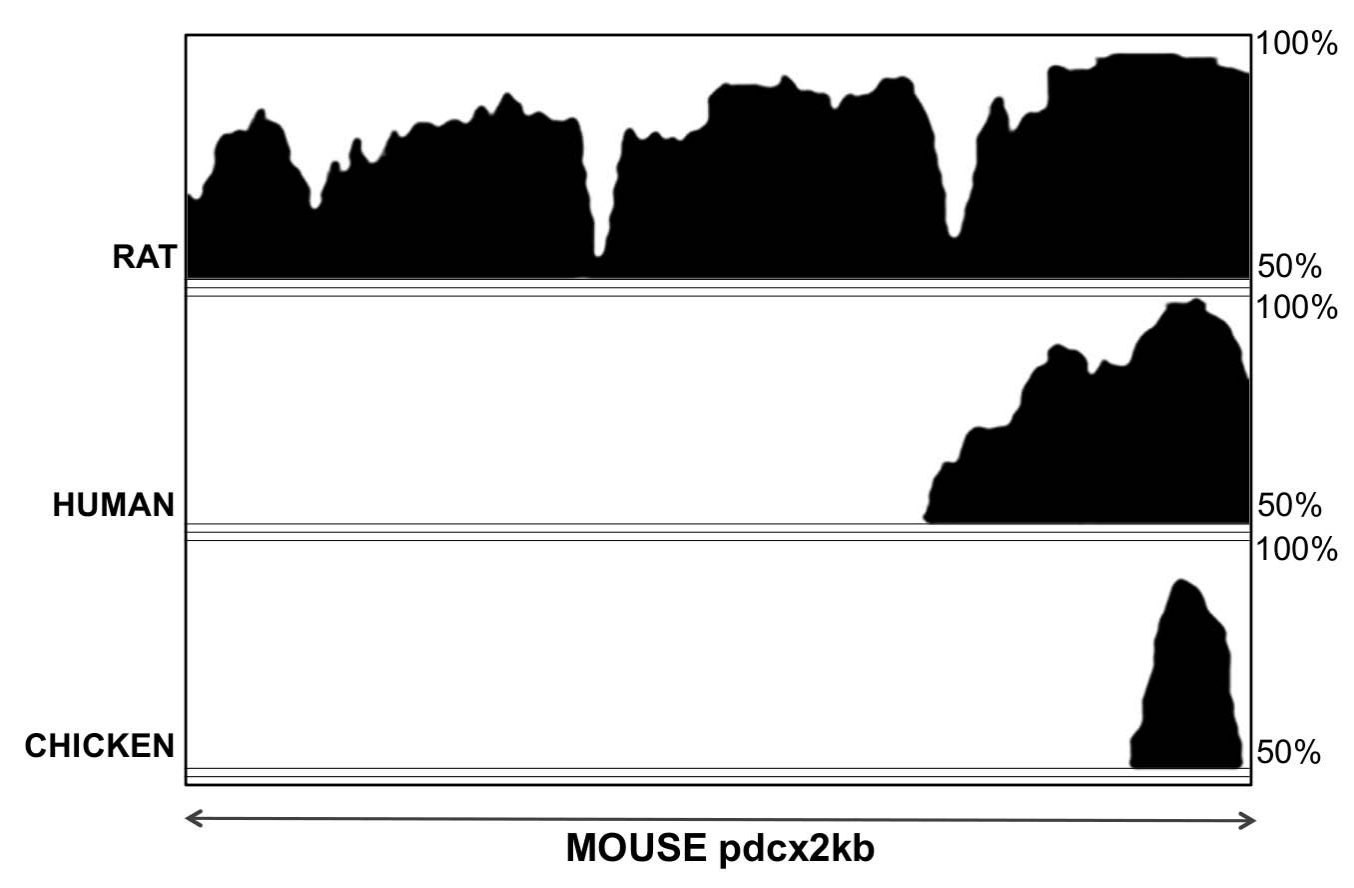
**A 183 bp region is highly conserved between different amniotes species**. Genomic sequences of mouse, rat, human and chick *pdcx *were compared using the VISTA http://www-gsd.lbl.gov/VISTA/ software. The positions of the sequences are shown on the horizontal axis and the percent identities (50-100%) are indicated in the vertical axis.

**Figure 6:**
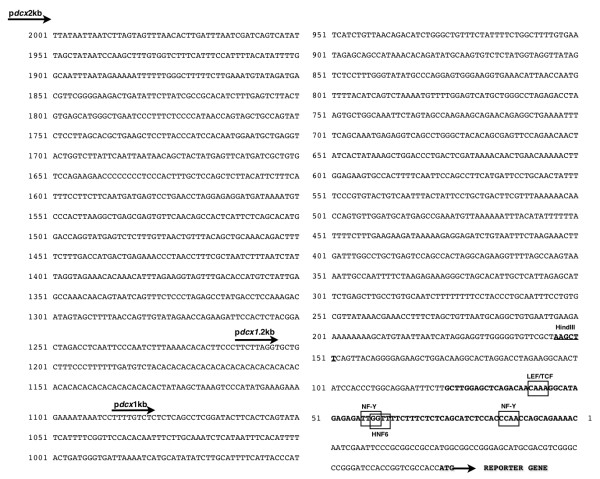
**Nucleotide sequence of the mouse *Dcx *gene regulatory sequence**. The fragments corresponding to *pdcx*2kb, *pdcx*1.2kb, *pdcx*1kb (obtained by PCR) and *pdcx*249bp (obtained by enzymatic restriction) are represented. Sequence of 79 bp Δ(-1820 -1741 from ATG of *Dcx *gene) is indicated in bold and putative binding sites for transcription factors NF-Y, HNF6 and LEF/TCF surrounded by boxes. Reporter gene translation Initiation Site is highlighted in bolt.

Comparative analyses were performed on the sequences of mouse, rat, human and chicken 2kb-long *Dcx *promoter fragments using the VISTA software (Figure 5). A high sequence similarity was found between mouse and rat *pdcx*2kb sequences. Comparison between mouse and human sequences revealed that sequence conservation was not detected throughout the 2kb-long sequence, but was limited to a ± 500 bp portion located at the *pdcx*2kb 3'-end. Similarly, sequence conservation between the mouse *pdcx*2kb and the chicken *pdcx*2kb was only observed in a 183bp-long fragment located at the 3'-end. In addition, the strongest sequence conservation between mouse, rat, human and chicken *pdcx*2kb sequences was detected within this 183bp-long fragment (Figure 5). Further analysis, using the MatInspector software, revealed that many putative binding sites for members of several transcription factor families known to participate in neuronal differentiation/migration such as NEUROD, NEUROG, BRN, PAX, HOX, SOX and DLX are present in the 249bp-, 1kb-, 2kb-long *Dcx *promoter fragments (Additional file 1: Table S1). In particular, this analysis revealed four conserved consensus binding sites for known transcription factors in the 79b-long fragment located at the 3'end of the *Dcx *promoter (Figure 6): two perfect consensus sites for NF-Y/CAAT, one perfect site for LEF/TCF which are effectors of the canonical Wnt pathway and one imperfect site for HNF6/OC2 (75% conserved relative to consensus), members of the ONECUT family [23-25].

### Expression of putative transcription factors during neuronal differentiation of mESC

**Figure 7:**
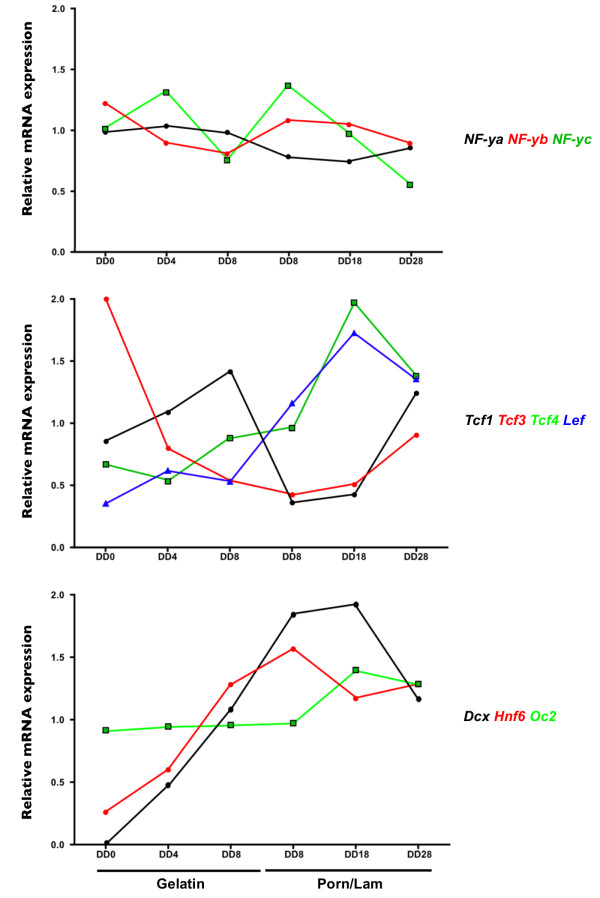
**Expression of transcription factors predicted to bind the *Dcx *regulatory sequence in the 3'-end region**. The relative abundance of mRNA coding for *Dcx, Lef, Tcf1, Tcf3, Tcf4, Hnf6, Oc2 *and *Nf-ya, Nf-yb and Nf-yc *were determined by semi-quantitative PCR at DD0, DD4 and DD8 on gelatin and at day of differentiation DD8, DD18 and DD28 on poly-ornithine/laminin. *Gapdh *was used as internal control.

To investigate which of these transcription factors, present in the 79 bp fragment and potentially involved in *Dcx *gene regulation, are expressed during neuronal differentiation of ESR1 cells, the levels of mRNA coding for *Dcx *and for each factor (*Lef, Tcf1, Tcf3 and Tcf4, Hnf6 *and *Oc2, Nf-ya, Nf-yb and Nf-yc*) were measured at different time-points during neuronal differentiation (Figure 7).

The abundance of each transcript at each stage was calculated relative to the *Gapdh *housekeeping gene mRNA, based on published results [26-28]. The observed pattern of *Dcx *transcript expression is consistent with that of Dcx protein synthesis during neuronal differentiation of ESR1 cells and with the observed transcriptional activity of the *Dcx *promoter (see above). No *Dcx *transcript was detected in undifferentiated ESR1 cells. Levels of *Dcx *transcripts progressively increased to reach a maximum at DD18 followed by a reduction at DD28. Each transcription factor transcript was detected in ESR1 cells. The temporal profile of *Lef*, *Hnf6 *and *Tcf4 *transcription factors presented patterns of expression similar to that of *Dcx*, suggesting that expression of these transcription factors is also dependent on neuronal differentiating time. Their levels were relatively low during the early phase of ESR1 cell differentiation (DD0-8), progressively increased during the late phase of the differentiation program to peak at DD18 when *Dcx *transcript levels were maximum. In contrast, *Nf-ya*, *Nf-yb*, *Nf-yc *and *Oc2 *transcripts displayed relatively constant steady state levels, with little variation during the entire differentiation program. The relative amounts of *Tcf1 *and *Tcf3*, seem to depend on the differentiation process, with *Tcf3 *decreasing during differentiation [29] and *Tcf1 *being apparently sensitive to the culture matrix substrate.

Since mRNA for each transcription factor was present in ESR1 cells and since no transcription factor could be discarded on the sole basis of the temporal expression profile of its transcript, selective mutagenesis experiments were undertaken.

**Figure 8:**
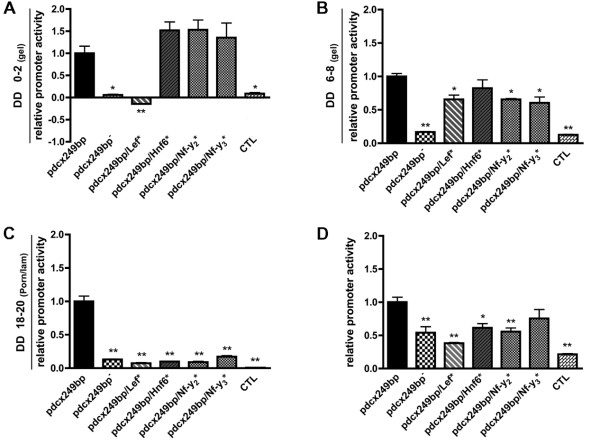
**Importance of individual binding site for *Dcx *promoter activity**. (**a-b-c**) ESR1 cells were transfected with the indicated constructs at day of differentiation 0, DD0 **(a)**, DD6 **(b) **on gelatin and at DD18 **(c) **on poly-ornithine/laminin and luciferase activity relative to β-galactosidase activity was determined 48 hours later. The different constructs were *pdcx*249bp-Luc, 79bp-deleted *pdcx*249bp-Luc construct (*pdcx*249bp^-^-Luc) and *pdcx*249bp-Luc with mutated binding site (*pdcx*249bp/Lef*, *pdcx*249bp/Hnf6*, *pdcx*249bp/Nf-y2* and *pdcx*249bp/Nf-y3*) and the promoterless control (CTL). **(d) **CGN were isolated at post-natal day 3 and were transfected the same day with the indicated constructs and luciferase activity relative to β-galactosidase activity was determined 48 hours later. The different constructs were *pdcx*249bp-Luc, 79bp-deleted *pdcx*249bp-Luc construct (*pdcx*249bp^-^-Luc) and *pdcx*249bp-Luc with mutated binding site (*pdcx*249bp/Lef*, *pdcx*249bp/Hnf6*, *pdcx*249bp/Nf-y2* and *pdcx*249bp/Nf-y3*) and the promoterless control (CTL). The activity of each construct is expressed relative to that of the *pdcx*249bp-Luc construct, set arbitrarily to 1. Note that the relative activity of *pdcx*249bp-Luc, used here as a reference was consistent with the results shown in figure 2. Each value represents the mean ± SEM of at least three independent transfection experiments, each performed in triplicate. Asterisks mean significantly different from *pdcx*249bp values at *P *< 0.05 (*) or *P *< 0.01 (**).

To determine the relative importance of each DNA binding site present in the 79bp-long *pdcx*-luciferase fragment, we individually altered each putative binding site for LEF/TCF, HNF6/OC2 or NF-Y/CAAT in the *Dcx *promoter using site-directed mutagenesis to generate *pdcx*249bp/Lef*, *pdcx*249bp/Hnf6* and *pdcx*249bp/Nf-y* (Figure 8). Then we compared the luciferase activities induced by each construct to that of *pdcx*249bp by transient expression experiments in ESR1 cells at three stages of neuronal differentiation. At the beginning of the differentiation program (DD2), mutation of the NF-Y or HNF6 binding sites did not affect *pdcx*249bp activity (Figure 8a). In contrast, mutation of the LEF/TCF site completely inhibited *pdcx*249bp activity. Such inhibition was not detected in ESR1 cells at DD8 (Figure 8b). At that stage, the relative activity of every mutated *pdcx*249b was below that of the wild type *pdcx*249bp but higher than that of the 79bp-truncated *pdcx*249bp. Finally, at DD20, when many ESR1 cells expressed the *Dcx *gene and displayed a neuronal phenotype, mutation of any of the four binding sites reduced the activity of *pdcx*249bp to that of the 79bp-truncated *pdcx*249bp (Figure 8c). Similar results were found using *pdcx*1kb (data not shown) and in PND3 cerebellar cells (Figure 8d). Simultaneous mutation of all three binding sites was also performed (*pdcx*249bp/Lef*/Hnf6*/Nf-y*). Transfection experiments revealed that the transcriptional activation of p*dcx*249bp mutated for LEF, HNF6 and NF-Y binding sites is similar to the constructs with any one mutated site (Additional file 2: Figure S1) at all stages, suggesting that an additional, not yet identified factor is responsible for the observed residual activity at DD8. Altogether, these results demonstrate first of all the importance of LEF/TCF, HNF6/OC2 or NF-Y/CAAT DNA binding sites for *Dcx *promoter activity at various stages of neuronal differentiation and that the corresponding factors are part of the regulatory mechanisms controlling *Dcx *promoter activity. However, these transcription factors are not sufficient to induce full transcriptional activation of the *Dcx *gene. In addition, the results obtained with the LEF/TCF binding site reveal that this binding site could be more specifically involved in activating *pdcx *at early stages of neuronal differentiation of ESR1 cells.

## Discussion

This present study shows that 249bp-, 1kb-, 2kb-long DNA fragments located upstream of the *Dcx *coding sequence, are sufficient for *in vitro *specific *Dcx *expression: in heterogeneous differentiated neurons from mouse embryonic stem (ESR1) cells, in primary mouse cerebellar neurons (PND3) and in organotypic slice cultures. The regulatory activity of all three constructs, visualized by eGFP expression, overlapped endogenous expression of Dcx or βIII-Tubulin (data not shown) in immature neuronal cells. In addition, no eGFP expression was observed in non-neuronal cells. These results strongly suggest that the fragments isolated here are sufficient for specific neuronal expression in differentiating neural cells.

During our analysis of transcriptional activities of the deletion constructs, we observed a very weak activity of the 1.2 kb fragment relative to the 1 kb construct, suggesting that the 0.2 kb sequence upstream from *pdcx*1kb could contain transcriptional repressor elements. To determine the regulatory element(s) susceptible to participate in transcriptional repression of *Dcx *expression, we analyzed this sequence with the MatInspector software http://www.genomatix.de, but no unique repressor element was identified. However, this 0.2 kb sequence holds a short tandem repeat (STR) of twenty-five CA repeats (Figure 6), that could act as positive or negative regulator of gene expression. Short tandem repeats have been identified and are widespread in coding and non-coding regions of eukaryotic genomes from yeast to humans [30,31]. CA repeats are also potential Z-DNA-forming sequences that could affect gene expression. Indeed, STRs were shown to activate or repress gene expression depending on the length of the repeats [32,33]. This potential repressive element was not further investigated in this work, but it certainly deserves further attention also in the context of *Dcx*-related disease.

Dcx is expressed in differentiating/migrating immature neurons of embryonic and adult CNS and PNS. Moreover, Dcx presents a maximum expression during corticogenesis (E14-E18 in mouse) and neurogenesis [18,34]. Using a mouse ESC model of neuronal differentiation, we demonstrated that the transcriptional activity of the *Dcx *promoter increased during the differentiation program, with a maximum activity shortly after the cells were plated on poly-ornithine/laminin substrates [35]. Both of these extracellular matrix (ECM) components were shown to promote the active extension of neuronal cell protrusions as well as their maturation [36-38]. In the present study, we further show that ECM components also promote expression of the endogenous Dcx protein, in a pattern fully consistent with the transcriptional activity of the identified promoter region during the same differentiation process. This interesting observation suggests that it is possible to reproduce the *Dcx *expression profile in an *in vitro *cell culture model and use it to better study the mechanisms involved in neuronal differentiation of ESC [1,2,5,34,39,40].

DNA binding sites for several transcription factors (E2F, NeuroD1, Brn2,..), known to participate to neuronal and migration processes were pointed out by bioinformatics analyses of the 2kb-long DNA regulatory sequence of the mouse *Dcx *gene. Most of the transcription factor binding sites present on the mouse DNA regulatory sequence were also detected on a 3.5-kb DNA fragment upstream of the translation start codon of the human *DCX *gene [41]. This human sequence included a sequence homologous to the sequence used in the present study and the first exon of the human *DCX *gene. Furthermore, deletion experiments revealed the presence of critical regulatory sequences in a short fragment showing high sequence homology across species. Within this sequence, three relevant consensus binding sites were detected. Each transcription factor selected in the *pdcx *3'-end (Lef/Tcf, Hnf6/Oc2, Nf-y/CAAT) is known to play a role, in a time-dependent manner, in the neuronal differentiation program. Mutational analysis revealed the outstanding importance of the LEF/TCF potential binding site for activation in early stages of the differentiation process, while at later stages the activity seems to be evenly distributed among all the binding sites. Simultaneous mutation of all three binding sites located in the 79 bp did not abolish the transcriptional activity of the *Dcx *promoter, implying that *Dcx *residual expression could be caused by a not yet discovered transcription factor located in the 79 bp region. Interestingly, the three transcription factors whose expression pattern during neuronal differentiation correlated best with *Dcx *promoter activity were Hnf6, Tcf4 and Lef. The latter are mediators in the canonical Wnt pathway that participates in many developmental processes *in vivo *and *in vitro *[42-46]. The involvement of *Lef *in the first steps of *Dcx *gene activation is consistent with the observations that some effectors of the Wnt canonical pathway contribute to neuronal differentiation of pluripotent cells, at least at specific stages [45,47]. Hirabayashi and co-workers proposed that the Wnt signaling pathway directs neuronal differentiation in the developing mouse neocortex [48]. They showed that activated β-catenin induced differentiation of Neural Precursor Cells (NPCs) at specific stages, whereas other studies suggested that Wnt signaling promoted NPC self-renewal [49]. LEF/TCF and the other identified transcription factor complexes also play a role at later stages of the neuronal differentiation of ESC.

*In silico *analysis of the *Dcx *promoter, combined with mutagenesis experiments and RT-PCR analyses singled out the interesting ONECUT factors. First, a putative binding element for HNF6/OC2 was identified (75% conserved relative to consensus) in the 79 bp sequence (four on the total sequence of p*dcx*2kb); second, the *Hnf6 *mRNA expression profile matched that of *Dcx *and third, mutation of the HNF6 binding site abolished *Dcx *promoter activity. Together, these results suggest that ONECUT factors could be involved in *Dcx *regulation. Hnf6 and other ONECUT factors have been detected in mouse [23,24], the zebrafish [50] and also in drosophila and ascidia developing nervous system [51,52]. All this information suggests that HNF6 and other members of the ONECUT family could play a role during neuronal differentiation and possibly in *Dcx *regulation. Further experiments are currently underway in order to clarify ONECUT action on *Dcx *expression

Data presented in this study suggest that the maturation state of differentiating neuroblasts could be characterized by a specific transcriptional activity of the *Dcx *gene. Some data suggest that the mouse embryonic brain (E14) contains two distinct populations of Dcx-positive cells, according to their Dcx expression levels [3]. In these Dcx-positive cells, isolated by flow cytometry from the brains of transgenic mice expressing *pdcx*-eGFP in embryonic, early postnatal and adult animals, cells with a low level of Dcx expression (Dcx^low^) were multipotent as shown by their expression of nestin. On the other hand, Dcx^high ^cells showed an established neuronal specification, characterized by their expression of neuronal markers like βIII-Tubulin or MAP2. These results support the idea that all Dcx^+ ^neuronal precursors do not have the same potential to differentiate. In this study, neuronal cells obtained from differentiation of ESC or primary cell cultures from brain embryos form a heterogeneous neuronal cell population. In this context, it is not surprising that Dcx^+ ^cells, obtained from ESC differentiation, display different levels of *Dcx *promoter activity. This most probably reflects an asynchrony of differentiating ESC stages in the same cell culture.

## Conclusions

The present work provides a molecular and cellular study of regulatory sequences (*pdcx*2kb, *pdcx*1kb, *pdcx*249bp), sufficient to promote specific neuronal expression of mouse *Dcx *in neuroblasts. We show here that *Dcx *expression is abolished when a specific region in the 3'-end of the promoter is lacking. In addition transcription factor binding sites (LEF/TCF, HNF6/OC2 and NF-Y/CAAT) localized in this promoter region seem to act in a time-dependent fashion on the transcriptional activity of the *Dcx *gene during neuronal differentiation of ESC. Future experiments on these short regulatory sequences will help to understand the transcriptional regulation of *Dcx *gene expression and, maybe to isolate precursors at defined stages of neuronal differentiation based on their *Dcx *expression.

## Methods

### Animals

All experimental procedures on animals were carried out according to the European Communities Council Directive (86/609/EEC) for care and use of Laboratory animals. The experimental protocols were reviewed and approved by the institutional Animal Care Committee.

### Materials

ES cell culture medium ingredients (DMEM/F12, Neurobasal medium, B27 supplement, Bovine Serum Albumin and L-glutamine) were obtained from Invitrogen-Gibco (Merelbeke, Belgium); BMP4 (Bone Morphogenic Protein-4) was obtained from R&D Systems (Abingdon, United Kingdom); apo-transferrin, progesterone, insulin, putrescine, sodium selenite, laminin, poly-ornithine and all chemicals were obtained from Sigma-Aldrich (St. Louis, MO, USA) unless specified otherwise and ESGRO^® ^(LIF, Leukemia Inhibitory Factor) was from Chemicon International (Temicula, CA, USA) unless specified otherwise.

### ESC Culture - Monoculture Differentiation and Transfection

ESR1 cells were maintained without feeder cells in serum-free culture medium. ESC were plated onto 0.1% gelatin-coated plates in N2B27 medium (as described by Ying et al., 2003 [28]) supplemented with ESGRO^® ^(LIF) 10^3 ^Units and BMP4 10 ng/ml and incubated at 37°C in 5% CO_2_. The medium was renewed every day. Cells were plated every 3 days using a dissociation solution (trypsin 0.05%, chicken serum 1%, EDTA 0.53 mM). To start monolayer differentiation, corresponding to DD0, undifferentiated cells were dissociated and plated onto 0.1% gelatin-coated plates at 10^4^cells/cm^2 ^in N2B27 medium supplemented with ESGRO^® ^(LIF) 10^3 ^Units. The medium (non-supplemented N2B27) was changed every other day during 8 days, until DD8. Then, at DD8, cells were dissociated and plated on poly-ornithine (100 μg/ml) and laminin (0.5 μg/ml)-coated plates. The medium was changed 2 h and 24 h after plating and every 2 days until 20 days of differentiation on porn/lam substrate, corresponding to DD28. ESR1 cells can differentiate into neural precursors, which could generate functional neurons [53], astrocytes, and oligodendrocytes [21,22].

Transient transfection experiments were performed using FuGene-6 (Roche Applied Science, Roche, Mannheim, Germany) as described by the manufacturer. eGFP expression was checked 24 h, 48 h and 72 h after transfection or luciferase/β-galactosidase activity ratios were determined 48 hours after transfection. The luciferase activity was measured according to the Luciferase Assay System (from Promega, Leiden, The Netherlands) and is expressed in RLU (Relative Light Units). The β-galactosidase activity was determined by reacting 40 μl of lysate with 40 μl of β-galactosidase substrate containing ONPG (o-nitrophenyl-D-galactopyranoside) and the absorbance was measured at 420 nm.

### Cerebellar Granule Neuron (CGN) Culture and Transfection

Primary cultures of CGNs were obtained from post-natal day 3 (PND3) 129/sv mice. Isolated cerebella were stripped off meninges in DPBS (Dulbecco's Phosphate-Buffered Saline) (Invitrogen-Gibco) supplemented with glucose (0.45%), minced, and treated with dissociation solution (0.25% trypsin, 0.01% DNase) for 25 min at 37°C. Cell dissociation was stopped by adding 0.5 ml of FBS (Fetal Bovine Serum) to the dissociation solution. Granule cells were then mechanically dissociated by two successive triturations and sedimentation steps in complete medium (DMEM, 25 mM KCl, 96 mM NaCl, 5 μM insulin, 2 mM glutamine, 1.5% glucose 30%, 1% sodium pyruvate, 5% heat-inactivated FBS, 10% horse serum), and resuspended in 3 ml of complete medium. Neurons were plated onto poly-ornithine (100 μg/ml)-coated 24-well culture plates (Nunc, Wiesbaden, Germany) at a density of ~1 × 10^4 ^cells/drop and incubated at 37°C in 5% CO_2_. The following day, transient transfection experiments were performed using FuGene-6 (Roche Applied Science) as described by the manufacturer. eGFP expression was checked 24 h, 48 h and 72 h after transfection and the cells were fixed for immunocytochemistry analysis or lysed for luciferase/β-galactosidase activity ratio determination 48 hours after transfection.

### Immunocytochemistry (ICC) and Immunohistochemistry (IHC)

Glass coverslips were washed in nitric acid (65%) for 1-2 days, rinsed in water for 2-3 hours, then in ethanol 100% for 2 h, air-dried and sterilized by autoclaving. Cells were washed in TBS (Tris-Buffered Saline), fixed for 20 min in 4% PFA (paraformaldehyde) at 4°C, washed in TBS and permeabilized/blocked for minimum 1 h in blocking buffer (TBS, 1% BSA, 0.2% teleostean gelatin, 0.1% Triton-X100) at room temperature [5]. For Immunohistochemistry, the blocking buffer was composed of TBS, 0.25% gelatin, 0.1% Triton-X100. The same buffer without Triton X100 was used for antibody incubations and washes. Cells were incubated overnight in primary antibody solution (Table 1) at 4°C (in humid chamber), washed 3 times and incubated for 1 h in secondary antibody solution at room temperature (in humid chamber). The latest wash was performed in TBS before mounting in Vectashield HardSet Mounting Medium with DAPI (Vector Laboratories, Peterborough, United Kingdom).

**Table 1:** Antibodies used for immunohistochemistry and Western blot analysis.

Antibody	reference	Dilution			Company
		**ICC**	**IHC**	**WB**	
Anti-Dcx(C-18)	(Goat) sc-8066	1/200	1/500	1/1000	SantaCruz Biotechnology
Anti-Oct3/4 (C10)	(Mouse) sc-5279	1/200		1/200	SantaCruz Biotechnology
Anti-βIII Tubulin	(Mouse) T8660	1/1000			Sigma-Aldrich
Anti-α-Tubulin	(Mouse) T6074			1/25000	Sigma-Aldrich
Anti-NeuN	(Mouse) MAB377	1/100	1/500		Chemicon
TRITC-conjugated	(DAG) 705-025-147	1/500	1/500		Jackson Immuno Research
	(DAM) 705-205-151	1/500	1/500		Jackson Immuno Research
HRP-conjugate	(RAG) A5420			1/2000	Sigma-Aldrich
	(MAG) A4416			1/2000	Sigma-Aldrich

### Western Blotting (WB)

After 2 washes with PBS, cells on 10 cm^2 ^plates were lysed by adding 300 μl lysis buffer (0.15 M NaCl, 0.05 M TrisBase, 1% TritonX-100, 1% Sodium deoxycholate, 0.1% SDS) supplemented with protease inhibitor cocktail (Roche) and PMSF (phenylmethylsulfonyl fluoride). After centrifugation at 4°C, the supernatant was removed and the protein content was determined using Bradford reagent (Bio-Rad Laboratories, Hercules, CA, USA). Samples were boiled in sample buffer (2×: 4% SDS, 10% β-mercaptoethanol, 135 mM Tris-HCl pH6.8, 20% glycerol, 1% bromophenol blue). Proteins (20 μg/sample) were separated by 10% SDS-PAGE, and transferred onto PVDF (Amersham Hybond-P). Blots were blocked in TBST-BSA 1% and incubated overnight with the primary antibody at 4°C. The following day, membranes were washed and then incubated 1 h with the secondary antibody at room temperature before washes. Detection was performed using ECL Plus method (Amersham).

### Plasmid Construction

The *Mus musculus *Doublecortin cDNA sequence is available under GenBank Accession no. NM010025 and genomic DNA sequence available under GenBank Accession no. BX530055, (Sanger Institute, Cambridge United Kingdom).

Bacterial Colony PCR-based screening was performed on the Library clone RP23-377E2 Bacterial Artificial Chromosome (BAC) (Children's Hospital Oakland Research Institute, California), using the Elongase Enzyme Mix (Invitrogen-Gibco) and three different 5'-primers (*pdcx *forward F1, F1.2, F2) and one 3'-primer (*pdcx *reverse):

*pdcx *F 1: 5'-TTTGTCTCTCTCAGCCTCGG-3'

*pdcx *F 1.2: 5'-TTCTTAGGTGCTGCTTTCCC-3'

*pdcx *F 2: 5'-ACTGACCTCTGTTCAGTTCC-3'

*pdcx *R: 5'-GTTTTCTGCTGGTTGGGTG-3'

Each PCR product (1 kbp, 1.2 kbp, 2 kbp) was cloned into pGEM-T easy (Promega) and subjected to DNA sequencing with T7 and SP6 oligonucleotide primers (GIGA, University of Liège, Belgium). The derived sequence was confirmed by comparison with the mouse genomic DNA obtained from C57Bl/6J mice and available under NCBI nucleotide bank Accession no. BX530055. Different fragments were cut with *Pst1*, *HindIII (*for *pdcx*249bp) and *Apa1 *restriction enzymes (New England Biolabs, Ipswich, MA, USA) and cloned into the pEGFP1 vector (BD/Clontech, Heidelberg, Germany). Integrity of the putative *Dcx *gene regulatory sequences of *Dcx *gene, *pdcx*-(1 kbp, 1.2 kbp, 2 kbp)-eGFP was confirmed by sequencing (Génome Express, Meylan, France).

### (RT)-PCR

Total RNA was extracted from cells (at day 0, 4, 8 on gelatin and day 0, 10 and 20 on poly-ornithine/laminin of ESC differentiation program, corresponding to DD0, DD4, DD8, DD18 and DD28 respectively) using instapure solution (Roche Applied Science, Vilvoorde, Belgium). Total RNA (5 μg) was used for a reverse transcriptional reaction. PCR was then carried out in a final volume of 50 μl containing 2.5 U of Taq polymerase (Promega), 2 μl of each selected primer and 0.2 μl of *Gapdh *primers (10 μM each, from Eurogentec, Seraing, Belgium), 1 μl of dNTP mix (10 mM each, from Promega) and 1 μl of the RT product. The primers used are detailed in Table 2.

**Table 2:** Primers used for PCR.

Gene	Sense primer (3' - 5')	Antisense primer (3' - 5')	Tm (°C)	Ref
***Dcx***	AAGTGACCAACAAGGCTATT	TCATTGTGTTTTCTCCCGGA	60	[54]
***Lef***	GAACGAGTCTGAAATCATCC	GTAGGGATATCAGGAGCTGG	53	[55]
***Tcf1***	CATTCCTGGAGTCCTGAAGC	CCTGTCAGTTACACCAACG	57	/
***Tcf3***	AGGAAATCACCAGTCACCGT	GTACTTGGCCTGTTCTTCTC	55	[56]
***Tcf4***	TTCAAAGACGACGGCGAACAG	TTGCTGTACGTGATAAGAGGCG	55	[56]
***Hnf6***	TTCCAGCGCATGTCGGCGCTC	GGTACTAGTCCGTGGTTCTTC	55	[57]
***Oc2***	ATGCCGGTCTCAGGGGACTCTC	GGCGAAGAGTGTTCGGCGTTGGAG	55	[58]
***Nf-ya***	CTGGAGCCTCTGATTGGGT	CTCTACAGATCCCAGGCAGC	55	[59]
***Nf-yb***	AGGATCCACCACCTTTTTGA	TAGCTGGGAGGCATCTGTG	55	[59]
***Nf-yc***	TTTCTTCCATGACTCTGGGC	GCTGCTTTCTTCGCTGGA	55	[59]
***Gapdh***	ACCACAGTCCATGCCATCAC	TCCACCACCCTGTTGCTGTA		

All sequences were designed using the PRIMER 3 software http://frodo.wi.mit.edu/primer3/input.htm.

PCR products were analyzed on agarose gel (Eurogentec) and quantified using Image Master 1D Prime v3.01 program (Amersham). The results are shown as the relative amount of the mRNA of interest relative to the *Gapdh *housekeeping gene mRNA.

### Site-directed mutagenesis

Site-directed mutagenesis of the LEF, HNF6 and NF-Y sites in the *Dcx *promoter was performed on *pdcx*249bp-luc plasmid. Whole plasmids carrying the mutation were obtained by performing PCR using primers listed in Table 3. Amplification reactions were carried out in 50 μl total volume containing 2.5 U of *Pfu *polymerase (Promega), 2 μl of each primer (10 μM each; Eurogentec), 1 μl of dNTP mix (10 mM each; Promega) and 50 ng DNA template (*pdcx*249bp-luc) and 2.5 units Pfu Ultra. The thermocycling program used was 94°C for 1 min, followed by 18 cycles of 94°C for 1 min, 55°C for 1 min, 72°C for 10 min and a final extension cycle of 72°C for 10 min. The template was degraded using 10 units of DpnI restriction endonuclease at 37°C for at least 2 h. After transformation of competent E. coli, recombinant plasmids were isolated and their sequence verified.

**Table 3:** Primers used for site-directed mutagenesis.

Mutated site	Primer (5'- 3')	Sequence	Ref
***pdcx***Lef*	Sense	ATCTCTCTATGCCGTGTTTGTCTGAGCTCCAAGC	[60]
	Antisense	GCTTGGAGCTCAGACAAACACGGCATAGAGAGAT	
***pdcx***Hnf6*	Sense	AGGCATAGAGAGCGTTTGTTTCTTTCTCTCAGCATCTCCACCCAACC	/
	Antisense	GGTTGGGTGGAGATGCTGAGAGAAAGAAACAAACGCTCTCTATGCCT	
***pdcx*****Nf-y**_1_*	Sense	AGGCATAGAGAGCGGTTTTTTCTTTCTCTCAGCTCTCCACCC	[61]
	Antisense	GGGTGGAGAGCTGAGAGAAAGAAAAAACCGCTCTCTAGCCT	
***pdcx*****Nf-y**_2_*	Sense	CGTTGTTTCTGCTGGTTTTGGGTGGAGATGC	[61]
	Antisense	GCATCTCCACCCAAAACCAGCAGAAACAACG	

PCR fragments carrying the mutation are obtained by annealing the following primers in the *pdcx*249bp-Luc plasmid.

### Ex vivo electroporation

Plasmids were prepared using the EndoFree Plasmid Kit (Qiagen, Hilden, Germany). E14.5 pregnant mice were anesthetized by gas and euthanized by cervical dislocation. The embryonic chain was removed from the mother, the embryos were isolated from the amniotic sac and decapitated. DNA was microinjected into lateral ventricles of isolated embryonic mouse heads placed in ice-cold L-15 medium supplemented with 3% glucose (1 M), 2.6% sodium bicarbonate (1 M) and 1% penicillin/streptomycin (100×). For DNA microinjection (using the FemtoJet apparatus, Eppendorf AG, Hamburg, Germany), 75-mm glass capillary tubes were pulled and tips were broken. Plasmid solutions were stained with Fast Green solution (0.05%) to monitor injection sites. Electroporations were performed on whole heads (skin and skull intact) using an ECM 830 electroporator (BTX) and the following parameters: five 50 ms long pulses separated by 1s long intervals at 50 V. After pulse delivery, the embryo heads were immersed in ice-cold L-15 supplemented with 3% glucose (1 M), 2.6% sodium bicarbonate (1 M) and 1% penicillin/streptomycin, brains were extracted and transferred into liquid 3% low melting agarose (37°C; Bio-Rad Laboratories, Hercules, CA, USA) and incubated on ice for 1 h. Embedded brains were cut coronally (250 μm) with a vibratome (VT1000S, Leica). Brain slices were transferred and maintained in organotypic slice cultures on sterilized culture plate inserts (0.4-μm pore size; Millicell-CM, Millipore Billerica, MA, USA). Brain slices were maintained in semi-dry conditions in wells containing Neurobasal medium supplemented with 2% B27, 1% N2, 1% penicillin/streptomycin. After 2 or 3 days, slices were fixed for 30 min in 4% PFA at 4°C and incubated overnight in sucrose solution (20%). The following day, slices were sectioned using a cryostat at 14 micron-thickness (LEICA CM3050S) and stained for immunofluorescence as previously described.

### Statistical analysis

Statistical analyses were performed by one-way ANOVA followed by Dunnett's post hoc tests, using a GraphPad Prism program (GraphPad, San Diego, CA, USA). Each experiment was performed in triplicates and repeated on at least three different occasions. Individual comparisons are expressed as mean ± SD.

## Abbreviations

Dcx: Doublecortin; CGN: Cerebellar Granule Neurons; DD: Day of Differentiation; BMP4: Bone Morphogenic Protein-4; GFAP: Glial fibrillary acidic protein; LIF: Leukemia Inhibitory Factor; MAP: Microtubule-Associated Protein; mESC: mouse Embryonic Stem Cells; PND: Post Natal Day; LEF1: Lymphoid enhancer factor-1; HNF6: Hepatocyte Nuclear Factor-6; OC2: Onecut-2; NF-Y: Nuclear Factor-Y

## Authors' contributions

MP carried out the study design, plasmid constructions, cell cultures, transfections, western blotting, Immunocytochemistry, *ex-vivo *electroporation, data analysis and drafted the manuscript. MM participated in the study design, sequence homologies and manuscript writing. MB performed the confocal immunofluorescence study and PCR experiments. GB performed the *pdcx*1kb-eGFP and *pdcx*1kb^-^-eGFP constructions. JCP participated in the study design and coordination and helped to draft the manuscript. All authors read and approved the final manuscript.

## Supplementary Material

Additional file 1 **Putative binding sites found in *Dcx *regulatory sequence of 2 kb**. These putative binding sites were detected using Matinspector software http://www.genomatix.de. Click here for file

Additional file 2 **Simultaneous mutation of the LEF, HNF6 and NF-Y binding sites in *Dcx *promoter**. ESR1 cells were transfected with the indicated constructs at day of differentiation 0 (DD0) and luciferase activity relative to β-galactosidase activity was determined 48 hours later. The different constructs were *pdcx*249bp-Luc, 79bp-deleted *pdcx*249bp-Luc construct (*pdcx*249bp^-^-Luc) and *pdcx*249bp-Luc with mutated binding site (*pdcx*249bp/Lef*, *pdcx*249bp/Hnf6*, *pdcx*249bp/Nf-y2*, *pdcx*249bp/Nf-y3* and *pdcx*249bp/Lef*/Hnf6*/Nf-y2*) and the promoterless control (CTL). The activity of each construct is expressed relative to that of the *pdcx*249bp-Luc construct, set arbitrarily to 1. Each value represents the mean ± SD of one transfection experiment, performed in triplicate. Asterisks mean significantly different from *pdcx*249bp values at *P *< 0.05 (*) or *P *< 0.01 (**). Click here for file
