# Supplementary material for: A short upstream promoter region mediates transcriptional regulation of the mouse doublecortin gene in differentiating neurons
Source: BMC Neurosci. 2010 May 28;11:64. doi: 10.1186/1471-2202-11-64 (PMC2891791; doi:10.1186/1471-2202-11-64)
Supplement: Additional file 1 — Putative binding sites found in Dcx regulatory sequence of 2 kb. These putative binding sites were detected using Matinspector software http://www.genomatix.de. [file 1471-2202-11-64-S1.PDF]

Matrix Family	Transcription factor:matrix family assignment<br>Detailed Family Information	Matrix	Position<br>from	to	strand	Core sim.	Matrix sim.	Sequence<br>red:ci-value>60<br>Capitals:core seq
V$BRNF	Brn POU domain factors	V$BRN2.01	8	26	(-)	0.933	0.895	ttAATTataaagctaggaa
O$VTBP	Vertebrate TATA binding protein factor	O$VTATA.01	8	24	(-)	1.000	0.905	aattaTAAAgctaggaa
V$CART	Cart-1 (cartilage homeoprotein 1)	V$S8.01	10	30	(-)	1.000	0.992	aagatTAATtataaagctagg
V$ATBF	AT-binding transcription factor	V$ATBF1.01	11	27	(+)	1.000	0.872	ctagctttatAATTaat
V$LHXF	Lim homeodomain factors	V$LHX1.01	11	33	(+)	1.000	0.917	ctagctttatAATTaatcttagt
V$PDX1	Pancreatic and intestinal homeodomain transcription factor	V$IPF1.01	11	29	(+)	1.000	0.908	ctagctttaTAATtaatct
V$DLXF	Distal-less homeodomain transcription factors	V$DLX1.01	11	29	(+)	1.000	1.000	ctagctttatAATTaatct
V$HNF1	Hepatic Nuclear Factor 1	V$HNF1.03	12	28	(-)	0.784	0.808	gATTAattataaagcta
V$LHXF	Lim homeodomain factors	V$LMX1A.01	12	34	(-)	1.000	0.927	tactaagattAATTataaagcta
V$NKXH	NKX homeodomain factors	V$NKX31.01	12	30	(-)	0.773	0.855	aagattAATTataaagcta
V$HOXF	Paralog hox genes 1-8 from the four hox clusters A, B, C, D	V$HOXC8.01	12	30	(-)	1.000	0.959	aagattaATTAtaaagcta
V$HBOX	Homeobox transcription factors	V$VAX2.01	13	31	(-)	1.000	0.940	taagattaATTAtaaagct
V$BRNF	Brn POU domain factors	V$BRN3.02	13	31	(+)	1.000	0.903	agctttaTAATtaatctta
V$HOMF	Homeodomain transcription factors	V$MSX1.01	13	31	(+)	1.000	0.951	agctttatAATTaatctta
V$BRNF	Brn POU domain factors	V$BRN3.03	14	32	(-)	1.000	0.924	ctaagatTAATtataaagc
V$HBOX	Homeobox transcription factors	V$VAX1.01	14	32	(+)	1.000	0.950	gctttatAATTaatcttag
V$OCT1	Octamer binding protein	V$OCT1.03	14	30	(+)	1.000	0.942	gctttataATTAatctt
V$HOMF	Homeodomain transcription factors	V$MSX1.01	14	32	(-)	1.000	0.954	ctaagattAATTataaagc
V$ARID	AT rich interactive domain factor	V$BRIGHT.01	14	32	(+)	1.000	0.951	gctttataATTAatcttag
V$ABDB	Abdominal-B type homeodomain transcription factors	V$HOXC9.01	15	31	(+)	1.000	0.849	ctttataaTTAAtctta
V$PAXH	PAX homeodomain binding sites	V$PAX4.02	15	29	(-)	1.000	0.895	agattAATTataaag
V$HOXF	Paralog hox genes 1-8 from the four hox clusters A, B, C, D	V$HOXC8.01	15	33	(+)	1.000	0.968	ctttataATTAatcttagt
V$NKX6	NK6 homeobox transcription factors	V$NKX63.01	15	29	(+)	1.000	0.892	ctttaTAATtaatct

V$OCT1	Octamer binding protein	V$OCT1.03	15	31	(-)	1.000	0.946	taagattaATTAtaaag
V$NKX1	NK1 homeobox transcription factors	V$NKX12.01	15	31	(+)	1.000	0.884	ctttatAATTaatctta
V$CART	Cart-1 (cartilage homeoprotein 1)	V$S8.01	15	35	(+)	1.000	0.997	ctttaTAATtaatcttagtag
V$PAXH	PAX homeodomain binding sites	V$PAX4.02	16	30	(+)	1.000	0.901	tttatAATTaatctt
V$PDX1	Pancreatic and intestinal homeodomain transcription factor	V$IPF1.01	16	34	(-)	1.000	0.919	tactaagatTAATtataaa
V$LHXF	Lim homeodomain factors	V$LMX1B.01	16	38	(-)	1.000	0.974	aaactactaagatTAATtataaa
V$NKX6	NK6 homeobox transcription factors	V$NKX61.01	16	30	(-)	1.000	0.956	aagaTTAAttataaa
V$DLXF	Distal-less homeodomain transcription factors	V$DLX2.01	16	34	(-)	1.000	0.977	tactaagattAATTataaa
V$BRNF	Brn POU domain factors	V$BRN4.02	17	35	(+)	1.000	0.831	ttataaTTAAtcttagtag
V$PIT1	GHF-1 pituitary specific pou domain transcription factor	V$PIT1.02	17	31	(-)	1.000	0.872	taagaTTAAttataa
V$PIT1	GHF-1 pituitary specific pou domain transcription factor	V$PIT1.02	18	32	(+)	1.000	0.843	tataaTTAAtcttag
V$NKXH	NKX homeodomain factors	V$NKX25.01	33	51	(-)	1.000	1.000	taaatcAAGTgttaaacta
V$PAX6	PAX-4/PAX-6 paired domain binding sites	V$PAX6.03	35	53	(+)	0.871	0.851	gtttaACACttgatttaat
V$PAX8	PAX-2/5/8 binding sites	V$PAX8.01	36	50	(-)	1.000	0.907	aaaTCAAgtgttaaa
V$LHXF	Lim homeodomain factors	V$LHX3.02	37	59	(+)	1.000	0.824	ttaacacttgattTAATcgatca
V$HNF6	Onecut homeodomain factor HNF6	V$OC2.01	39	55	(-)	1.000	0.845	cgattaAATCaagtgtt
V$CLOX	CLOX and CLOX homology (CDP) factors	V$CPHX.01	39	57	(+)	1.000	0.869	aacactTGATttaatcgat
V$HOMF	Homeodomain transcription factors	V$HHEX.01	39	57	(+)	1.000	0.959	aacacttgattTAATcgat
V$HBOX	Homeobox transcription factors	V$EN1.01	40	58	(+)	1.000	0.774	acacttgaTTTAatcgatc
V$PARF	PAR/bZIP family	V$TEF.01	40	56	(+)	0.773	0.864	acacttgatTTAAtcga
V$HOXC	HOX - PBX complexes	V$PBX_HOXA9.01	41	57	(+)	1.000	0.815	cactTGATttaatcgat
V$LHXF	Lim homeodomain factors	V$ISL2.01	42	64	(-)	1.000	0.883	atgactgatcgATTAaatcaagt
V$CLOX	CLOX and CLOX homology (CDP) factors	V$CLOX.01	43	61	(-)	1.000	0.937	actgATCGattaaatcaag
V$HOMF	Homeodomain transcription factors	V$HMX3.03	43	61	(+)	1.000	0.867	cttgattTAATcgatcagt

V$HNF6	Onecut homeodomain factor HNF6	V$OC2.01	45	61	(+)	1.000	0.828	tgatttAATCgatcagt
V$NKX6	NK6 homeobox transcription factors	V$NKX61.02	45	59	(+)	1.000	0.906	tgatTTAAtcgatca
V$CART	Cart-1 (cartilage homeoprotein 1)	V$PHOX2.01	45	65	(+)	1.000	0.872	tgattTAATcgatcagtcata
V$HOXC	HOX - PBX complexes	V$HOX_PBX.01	47	63	(-)	1.000	0.850	tgacTGATcgattaaat
V$HZIP	Homeodomain-leucine zipper transcription factors	V$HOMEZ.01	48	62	(-)	1.000	0.852	gactgATCGattaaa
V$CLOX	CLOX and CLOX homology (CDP) factors	V$CDPCR3HD.01	48	66	(+)	1.000	0.976	tttaatcGATCagtcatat
V$HOXH	HOX - MEIS1 heterodimers	V$MEIS1A_HOXA9.01	49	63	(-)	1.000	0.772	TGACtgatcgattaa
V$AP1R	MAF and AP1 related factors	V$TCF11MAFG.01	49	69	(-)	1.000	0.812	ctaataTGACtgatcgattaa
V$AP1R	MAF and AP1 related factors	V$BACH1.01	51	71	(+)	0.750	0.866	aatcgaTCAGtcatattagct
V$CART	Cart-1 (cartilage homeoprotein 1)	V$PHOX2.01	68	88	(+)	1.000	0.871	agctaTAATccaagctttgtg
V$BCDF	Bicoid-like homeodomain transcription factors	V$OBOX6.01	68	84	(+)	1.000	0.894	agctatAATCcaagctt
V$SORY	SOX/SRY-sex/testis determinig and related HMG box factors	V$SOX9.02	72	94	(-)	1.000	0.944	aaagaccACAAagcttggattat
V$NFAT	Nuclear factor of activated T-cells	V$NFAT5.01	86	104	(-)	1.000	0.864	aatGGAAatgaaagaccac
V$ETSF	Human and murine ETS1 factors	V$SPIB.01	89	109	(-)	1.000	0.892	tgtaaaatGGAAatgaaagac
V$OCT1	Octamer binding protein	V$OCT1.04	90	106	(-)	0.846	0.901	aaAATGgaaatgaaaga
V$IRFF	Interferon regulatory factors	V$IRF3.01	90	110	(-)	1.000	0.851	atgtaaaatgGAAAtgaaaga
O$INRE	Core promoter initiator elements	O$DINR.01	92	102	(+)	0.969	0.955	ttTCATttcca
V$YY1F	Activator/repressor binding to transcription initiation site	V$YY2.01	95	115	(+)	1.000	0.975	catttCCATtttacatatttt
V$OCT1	Octamer binding protein	V$OCT1.04	97	113	(-)	1.000	0.832	aaTATGtaaaatggaaa
V$CREB	cAMP-responsive element binding proteins	V$E4BP4.01	98	118	(-)	1.000	0.857	gccaaaatatGTAAaatggaa
V$BRNF	Brn POU domain factors	V$BRN2.01	99	117	(+)	1.000	0.871	tcCATTttacatattttgg
V$PARF	PAR/bZIP family	V$VBP.01	101	117	(-)	1.000	0.886	ccaaaatatGTAAaatg
V$SRFF	Serum response element binding factor	V$SRF.01	102	120	(-)	0.773	0.713	ttgccaaAATAtgtaaaat
V$FKHD	Fork head domain factors	V$FHXB.01	105	121	(-)	0.818	0.831	attgccAAAAtatgtaa

V$CEBP	Ccaat/Enhancer Binding Protein	V$CEBPB.01	109	123	(+)	1.000	0.942	atattttgGCAAttt
V$HOMF	Homeodomain transcription factors	V$HHEX.01	112	130	(+)	1.000	0.984	ttttggcaattTAATagaa
V$HBOX	Homeobox transcription factors	V$EN1.01	113	131	(+)	1.000	0.874	tttggcaaTTTAatagaaa
V$LHXF	Lim homeodomain factors	V$ISL1.01	114	136	(+)	1.000	0.828	ttggcaattTAATagaaaaattt
V$HBOX	Homeobox transcription factors	V$EN1.01	115	133	(-)	0.826	0.818	tttttctaTTAAattgcca
V$HOXH	HOX - MEIS1 heterodimers	V$MEIS1B_HOXA9.01	115	129	(+)	0.750	0.830	TGGCaatttaataga
V$ABDB	Abdominal-B type homeodomain transcription factors	V$HOXC13.01	117	133	(-)	1.000	0.952	tttttctatTAAAttgc
V$HOMF	Homeodomain transcription factors	V$HMX3.02	117	135	(-)	1.000	0.926	aatttttctaTTAAattgc
V$NKX6	NK6 homeobox transcription factors	V$NKX61.02	118	132	(+)	1.000	0.904	caatTTAAtagaaaa
V$CART	Cart-1 (cartilage homeoprotein 1)	V$PHOX2.01	118	138	(+)	1.000	0.877	caattTAATagaaaaattttt
V$BCL6	POZ domain zinc finger expressed in B-Cells	V$BCL6.02	119	135	(+)	1.000	0.821	aatttaaTAGAaaaatt
V$SATB	Special AT-rich sequence binding protein	V$SATB1.01	121	135	(+)	1.000	0.966	tttAATAgaaaaatt
V$FKHD	Fork head domain factors	V$FHXB.01	129	145	(-)	0.818	0.847	aagcccAAAAaatttt
V$ARID	AT rich interactive domain factor	V$JARID2.01	129	147	(+)	0.850	0.900	aaaaaTTTTttgggctttt
V$HEAT	Heat shock factors	V$HSF1.02	138	162	(-)	0.784	0.767	ctatacatttCAAGaaaaagcccaa
V$HEAT	Heat shock factors	V$HSF1.01	139	163	(+)	0.857	0.862	tgggctttttctTGAAatgtataga
V$CIZF	CAS interating zinc finger protein	V$NMP4.01	140	150	(-)	1.000	0.976	agAAAAagccc
V$BCL6	POZ domain zinc finger expressed in B-Cells	V$BCL6.02	143	159	(+)	0.771	0.833	ctttttcTTGAaatgta
V$OCT1	Octamer binding protein	V$OCT3_4.02	147	163	(-)	0.791	0.883	tctatACATttcaagaa
V$CLOX	CLOX and CLOX homology (CDP) factors	V$CLOX.01	150	168	(-)	0.807	0.848	cgtcATCTatacatttcaa
V$PIT1	GHF-1 pituitary specific pou domain transcription factor	V$PIT1.01	153	167	(-)	0.806	0.940	gtcatCTATacattt
V$CREB	cAMP-responsive element binding proteins	V$CREB.02	155	175	(+)	1.000	0.960	atgtatagaTGACgttcgggg
V$CREB	cAMP-responsive element binding proteins	V$ATF.02	158	178	(+)	1.000	0.932	tatagaTGACgttcggggaag
V$HIFF	Hypoxia inducible factor, bHLH/PAS protein family	V$HIF1.01	160	176	(-)	1.000	0.896	tccccgaACGTcatcta
V$STAT	Signal transducer and activator of transcription	V$STAT5.01	163	181	(-)	0.945	0.896	agtcTTCCccgaacgtcat

V$STAT	Signal transducer and activator of transcription	V$STAT.01	165	183	(+)	1.000	0.971	gacgttcggGGAAgactga
V$IKRS	Ikaros zinc finger family	V$IK3.01	169	181	(+)	1.000	0.866	ttcggGGAAgact
V$ZF35	Zinc finger protein ZNF35	V$ZNF35.01	170	182	(+)	1.000	0.979	tcggggAAGActg
V$PCBE	PREB core-binding element	V$PREB.01	174	188	(-)	1.000	0.864	gaataTCAGtcttcc
V$GATA	GATA binding factors	V$GATA2.01	185	197	(-)	1.000	0.961	cggcGATAagaat
V$PAX5	PAX-5 B-cell-specific activator protein	V$PAX5.01	187	215	(-)	0.905	0.801	gtaagaCTCAaagatgtgcggcgataaga
V$GZF1	GDNF-inducible zinc finger gene 1	V$GZF1.01	189	201	(-)	0.750	0.735	TGTGcggcgataa
V$PAX3	PAX-3 binding sites	V$PAX3.01	191	209	(+)	0.780	0.761	aTCGCcgcacatctttgag
V$EVI1	EVI1-myleoid transforming protein	V$EVI1.07	194	210	(-)	1.000	0.904	actcaAAGAtgtgcggc
V$LEFF	LEF1/TCF	V$LEF1.02	198	214	(-)	1.000	0.940	taagactCAAAgatgtg
V$RXRF	RXR heterodimer binding sites	V$VDR_RXR.03	235	259	(-)	1.000	0.768	cagctactGGTTatggggagaaagg
V$SNAP	snRNA-activating protein complex	V$PSE.02	240	258	(+)	0.893	0.760	ctcccCATAaccagtagct
V$HNF1	Hepatic Nuclear Factor 1	V$HMBOX.01	242	258	(-)	1.000	0.905	agctactgGTTAtgggg
V$GRHL	Grainyhead-like transcription factors	V$GRHL1.01	245	257	(-)	1.000	0.893	gctactGGTTatg
V$CP2F	CP2-erythrocyte Factor related to drosophila Elf1	V$CP2.02	247	265	(-)	1.000	0.862	tACTGgcagctactggtta
V$HEAT	Heat shock factors	V$HSF1.03	250	274	(+)	0.816	0.778	ccagtagctgccAGTAtctccttag
V$TALE	TALE homeodomain class recognizing TG motifs	V$TGIF2.01	251	267	(+)	0.754	0.811	cagtagctGCCAgtatc
V$GATA	GATA binding factors	V$GATA1.01	259	271	(-)	1.000	0.965	aggaGATActggc
V$AHRR	AHR-arnt heterodimers and AHR-related factors	V$AHRARNT.03	265	289	(-)	1.000	0.959	aggagcttcaGCGTgctaaggagat
V$RXRF	RXR heterodimer binding sites	V$VDR_RXR.04	276	300	(-)	0.750	0.814	gtggatgGGTAaggagcttcagcgt
V$FAST	FAST-1 SMAD interacting proteins	V$FAST1.02	290	306	(-)	1.000	0.816	tccatTGTGgatgggta
V$SORY	SOX/SRY-sex/testis determinig and related HMG box factors	V$SOX9.02	292	314	(+)	1.000	0.963	cccatccACAAtggaatgctgag
V$OCT1	Octamer binding protein	V$OCT1.05	295	311	(-)	0.900	0.891	agCATTccattgtggat
V$SORY	SOX/SRY-sex/testis determinig and related HMG box factors	V$SOX9.03	297	319	(-)	1.000	0.814	agtacctcagcattccaTTGTgg
V$TEAF	TEA/ATTS DNA binding domain factors	V$TEAD.01	300	312	(-)	1.000	0.932	cagCATTccattg
V$CREB	cAMP-responsive element binding proteins	V$CREB.03	305	325	(+)	0.793	0.873	gaatgcTGAGgtactggtctt

V$ZFTR	Zinc finger transcriptional repressor	V$ZNF217.01	305	317	(+)	1.000	0.927	GAATgctgaggta
V$RU49	Zinc finger transcription factor RU49, zinc finger proliferation 1 - Zipro1	V$RU49.01	314	320	(-)	1.000	0.994	cAGTAcc
V$CART	Cart-1 (cartilage homeoprotein 1)	V$CART1.01	319	339	(+)	0.798	0.898	tggtcTTATtcaattaataac
V$CART	Cart-1 (cartilage homeoprotein 1)	V$S8.01	319	339	(-)	1.000	1.000	gttatTAATtgaataagacca
V$LHXF	Lim homeodomain factors	V$ISL2.01	320	342	(+)	1.000	0.913	ggtcttattcaATTAataacagc
V$DLXF	Distal-less homeodomain transcription factors	V$DLX1.02	320	338	(+)	1.000	0.885	ggtcttattcAATTaataa
V$SORY	SOX/SRY-sex/testis determinig and related HMG box factors	V$HMGA.01	320	342	(+)	1.000	0.910	ggtcttattcAATTaataacagc
V$LHXF	Lim homeodomain factors	V$LHX5.01	321	343	(-)	1.000	0.892	agctgttattAATTgaataagac
V$HOXF	Paralog hox genes 1-8 from the four hox clusters A, B, C, D	V$HOXB4.01	321	339	(-)	1.000	0.871	gttattAATTgaataagac
V$NKXH	NKX homeodomain factors	V$NKX25.02	321	339	(-)	1.000	0.903	gttatTAATtgaataagac
V$BRNF	Brn POU domain factors	V$BRN2.04	322	340	(+)	0.763	0.842	tcttattCAATtaataaca
V$HOMF	Homeodomain transcription factors	V$HMX3.02	322	340	(+)	1.000	0.940	tcttattcaaTTAAtaaca
V$HBOX	Homeobox transcription factors	V$GSH2.01	322	340	(-)	1.000	0.957	tgttatTAATtgaataaga
V$CART	Cart-1 (cartilage homeoprotein 1)	V$RHOX6.01	323	343	(-)	0.784	0.847	agctgTTATtaattgaataag
V$HBOX	Homeobox transcription factors	V$MEOX1.01	323	341	(+)	1.000	0.854	cttattcAATTaataacag
V$OCT1	Octamer binding protein	V$OCT1.03	323	339	(+)	1.000	0.850	cttattcaATTAataac
V$NKX1	NK1 homeobox transcription factors	V$NKX12.01	323	339	(-)	1.000	0.875	gttattAATTgaataag
V$BCDF	Bicoid-like homeodomain transcription factors	V$PCE1.01	323	339	(-)	1.000	0.947	gttatTAATtgaataag
V$BRNF	Brn POU domain factors	V$BRN3.02	323	341	(-)	1.000	0.911	ctgttatTAATtgaataag
V$ARID	AT rich interactive domain factor	V$BRIGHT.01	323	341	(+)	1.000	0.954	cttattcaATTAataacag
V$HOMF	Homeodomain transcription factors	V$MSX.01	323	341	(-)	1.000	0.978	ctgttatTAATtgaataag
V$PDX1	Pancreatic and intestinal homeodomain transcription factor	V$PDX1.01	324	342	(+)	1.000	0.772	ttattcaatTAATaacagc
V$HNF1	Hepatic Nuclear Factor 1	V$HNF1.03	324	340	(-)	1.000	0.883	tGTTAttaattgaataa
V$LHXF	Lim homeodomain factors	V$LHX3.01	324	346	(+)	1.000	0.830	ttattcaaTTAAtaacagctact
V$CART	Cart-1 (cartilage homeoprotein 1)	V$UNCX4.01	324	344	(+)	1.000	0.873	ttattcAATTaataacagcta

V$HOXF	Paralog hox genes 1-8 from the four hox clusters A, B, C, D	V$HOXB8.01	324	342	(+)	1.000	0.892	ttattcaATTAataacagc
V$PAXH	PAX homeodomain binding sites	V$PAX6_HD.01	324	338	(-)	1.000	0.880	ttattAATTgaataa
V$HOXF	Paralog hox genes 1-8 from the four hox clusters A, B, C, D	V$HOXB8.01	325	343	(-)	1.000	0.868	agctgttATTAattgaata
V$DLXF	Distal-less homeodomain transcription factors	V$DLX3.01	325	343	(-)	1.000	0.941	agctgttatTAATtgaata
V$NKX6	NK6 homeobox transcription factors	V$NKX61.01	325	339	(-)	1.000	0.927	gttaTTAAttgaata
V$PIT1	GHF-1 pituitary specific pou domain transcription factor	V$PIT1.02	326	340	(-)	1.000	0.846	tgttaTTAAttgaat
V$BRNF	Brn POU domain factors	V$BRN3.03	326	344	(+)	1.000	0.905	attcaatTAATaacagcta
V$ATBF	AT-binding transcription factor	V$ATBF1.01	327	343	(-)	1.000	0.855	agctgttattAATTgaa
V$PIT1	GHF-1 pituitary specific pou domain transcription factor	V$PIT1.02	327	341	(+)	1.000	0.872	ttcaaTTAAtaacag
V$ABDB	Abdominal-B type homeodomain transcription factors	V$HOXC9.01	327	343	(-)	1.000	0.902	agctgttaTTAAttgaa
V$HOMF	Homeodomain transcription factors	V$HMX3.02	327	345	(-)	1.000	0.930	gtagctgttaTTAAttgaa
V$NKX6	NK6 homeobox transcription factors	V$NKX61.01	328	342	(+)	1.000	0.956	tcaaTTAAtaacagc
V$OVOL	OVO homolog-like transcription factors	V$OVOL1.01	331	345	(-)	1.000	0.821	gtagctGTTAttaat
V$MYBL	Cellular and viral myb-like transcriptional regulators	V$VMYB.01	334	346	(+)	0.817	0.898	aatAACAgctact
V$ABDB	Abdominal-B type homeodomain transcription factors	V$HOXC9.01	347	363	(-)	0.805	0.845	agcgatcaTGAActcat
V$HEAT	Heat shock factors	V$HSF1.02	358	382	(+)	0.811	0.782	atcgctgtgtCCAGaagaacccccc
V$ZBPF	Zinc binding protein factors	V$ZBP89.01	370	392	(+)	1.000	0.940	agaagaacccCCCCctcccactt
V$ZBPF	Zinc binding protein factors	V$ZNF219.01	372	394	(+)	1.000	0.988	aagaaccCCCCcctcccactttg
V$KLFS	Krueppel like transcription factors	V$KLF6.01	372	388	(-)	1.000	0.950	gggaGGGGgggttctt
V$GLIF	GLI zinc finger family	V$ZIC2.01	373	387	(+)	1.000	0.955	agaacccCCCCctcc
V$SPZ1	Testis-specific bHLH-Zip transcription factors	V$SPZ1.01	373	383	(-)	0.925	0.953	gGGGGggttct
V$MAZF	Myc associated zinc fingers	V$MAZR.01	374	386	(-)	1.000	0.939	gaggggGGGGttc
V$EGRF	EGR/nerve growth factor induced protein C & related factors	V$WT1.01	374	390	(-)	0.837	0.945	gtgggAGGGgggggttc
V$KLFS	Krueppel like transcription factors	V$KKLF.01	375	391	(-)	1.000	0.921	agtgggaGGGGggggtt

V$PURA	Pur-alpha binds both single-stranded and double-stranded DNA in a sequence-specific manner	V$PURALPHA.01	375	387	(-)	1.000	0.991	ggAGGGggggtt
V$EGRF	EGR/nerve growth factor induced protein C & related factors	V$CKROX.01	376	392	(-)	1.000	0.981	aagtGGGAgggggggt
V$SP1F	GC-Box factors SP1/GC	V$GC.01	377	391	(-)	0.877	0.894	agtgGGAGggggggg
V$INSM	Insulinoma associated factors	V$INSM1.01	377	389	(-)	1.000	0.921	tgggaGGGGgggg
V$MAZF	Myc associated zinc fingers	V$MAZ.01	378	390	(-)	1.000	0.951	gtggGAGGggggg
V$HOMF	Homeodomain transcription factors	V$HMX3.01	382	400	(-)	1.000	0.922	ctggagcaAAGTgggaggg
V$LEFF	LEF1/TCF	V$LEF1.01	385	401	(-)	1.000	0.869	gctggagCAAAgtggga
V$SORY	SOX/SRY-sex/testis determinig and related HMG box factors	V$HBP1.01	398	420	(-)	1.000	0.882	gaaatgaaagAATGtaagagctg
O$INRE	Core promoter initiator elements	O$DINR.01	412	422	(+)	0.969	0.959	ttTCATttcct
V$EVI1	EVI1-myleoid transforming protein	V$MEL1.02	424	440	(+)	1.000	1.000	cttcaatGATGagtcct
V$AP1R	MAF and AP1 related factors	V$BACH1.01	427	447	(+)	1.000	0.863	caatgaTGAGtcctgaaccta
V$NRSF	Neuron-restrictive silencer factor	V$NRSF.02	435	465	(+)	0.836	0.762	agtcctGAACctaggagaggatgataaaatg
V$PAX6	PAX-4/PAX-6 paired domain binding sites	V$PAX6.04	439	457	(-)	0.889	0.851	catCCTCtcctaggttcag
V$EVI1	EVI1-myleoid transforming protein	V$MEL1.03	447	463	(+)	1.000	0.962	aggagagGATGataaaa
V$DMRT	DM domain-containing transcription factors	V$DMRT1.01	450	470	(+)	0.934	0.771	agaggatgataaaATGTccca
V$CDXF	Vertebrate caudal related homeodomain protein	V$CDX1.01	451	469	(-)	1.000	0.942	gggacatTTTAtcatcctc
V$GATA	GATA binding factors	V$GATA1.03	453	465	(+)	1.000	0.951	ggatGATAaaatg
V$RORA	v-ERB and RAR-related orphan receptor alpha	V$RORA2.01	455	477	(-)	0.750	0.824	ccttaagtgGGACattttatcat
V$DMRT	DM domain-containing transcription factors	V$DMRT4.01	456	476	(-)	0.800	0.806	cttaagtggGACAttttatca
V$NKXH	NKX homeodomain factors	V$NKX32.01	461	479	(-)	1.000	0.969	agccttaAGTGggacattt
V$HOMF	Homeodomain transcription factors	V$HMX2.01	462	480	(+)	1.000	0.930	aatgtcccaCTTAaggctg
V$HOMF	Homeodomain transcription factors	V$HMX3.01	463	481	(-)	1.000	0.932	tcagccttAAGTgggacat
V$HOMF	Homeodomain transcription factors	V$HMX2.01	467	485	(-)	1.000	0.922	tcgctcagcCTTAagtggg

V$NKXH	NKX homeodomain factors	V$NKX32.01	489	507	(-)	1.000	0.964	gagaatgAGTGgctgtgaa
V$YBXF	Y-box binding transcription factors, multifunctional proteins involved in transcriptional and translational regulation, mRNA splicing, DNA replication and repair	V$YB1.01	491	503	(-)	1.000	0.906	atgagTGGCtgtg
V$SORY	SOX/SRY-sex/testis determinig and related HMG box factors	V$HBP1.01	492	514	(-)	1.000	0.867	atgtgctgagAATGagtggctgt
V$CAAT	CCAAT binding factors	V$CAAT.01	492	506	(+)	0.827	0.935	acagCCACtcattct
V$YY1F	Activator/repressor binding to transcription initiation site	V$YY1.02	501	521	(-)	1.000	0.951	ctggtCCATgtgctgagaatg
V$NRSF	Neuron-restrictive silencer factor	V$NRSF.02	503	533	(+)	1.000	0.739	ttctcaGCACatggaccaggtatgagtctct
V$HAND	Twist subfamily of class B bHLH transcription factors	V$PARAXIS.01	503	523	(+)	0.882	0.889	ttctcAGCAcatggaccaggt
V$MITF	Microphthalmia transcription factor	V$MIT.01	506	520	(-)	1.000	0.941	tggtcCATGtgctga
V$HESF	Vertebrate homologues of enhancer of split complex	V$DEC2.01	506	520	(+)	0.903	0.961	tcagcaCATGgacca
V$SORY	SOX/SRY-sex/testis determinig and related HMG box factors	V$SOX2.01	522	544	(-)	1.000	0.939	acagttaACAAagagactcatac
V$LEFF	LEF1/TCF	V$LEF1.01	527	543	(-)	1.000	0.880	cagttaaCAAAgagact
V$HNF1	Hepatic Nuclear Factor 1	V$HNF1.04	528	544	(+)	1.000	0.931	gtctctttGTTAactgt
V$OVOL	OVO homolog-like transcription factors	V$OVOL1.01	530	544	(+)	1.000	0.805	ctctttGTTAactgt
V$FKHD	Fork head domain factors	V$XFD3.01	530	546	(-)	1.000	0.887	aaacagttAACAagag
V$HOMF	Homeodomain transcription factors	V$HMX2.03	531	549	(+)	1.000	0.857	tctttgTTAActgtttaca
V$OVOL	OVO homolog-like transcription factors	V$OVOL1.01	533	547	(-)	1.000	0.821	taaacaGTTAacaaa
V$HNF1	Hepatic Nuclear Factor 1	V$HNF1.04	533	549	(-)	1.000	0.911	tgtaaacaGTTAacaaa
V$FKHD	Fork head domain factors	V$HNF3.01	537	553	(-)	1.000	0.983	cagctgtAAACagttaa
V$FKHD	Fork head domain factors	V$HNF3.01	548	564	(+)	1.000	0.995	cagctgcAAACagactt
V$HEAT	Heat shock factors	V$HSF2.02	556	580	(-)	1.000	0.961	agtcatggtcaaAGAAaagtctgtt
V$NR2F	Nuclear receptor subfamily 2 factors	V$HNF4.01	557	581	(-)	1.000	0.852	cagtcatggtCAAAgaaaagtctgt
V$RXRF	RXR heterodimer binding sites	V$LXRE.02	559	583	(-)	1.000	0.729	ctcagtcatGGTCaaagaaaagtct
V$PERO	Peroxisome proliferator-activated receptor	V$PPAR_RXR.02	560	582	(-)	1.000	0.729	tcagtcatggtcAAAGaaaagtc
V$LEFF	LEF1/TCF	V$LEF1.02	562	578	(-)	1.000	0.951	tcatggtCAAAgaaaag

V$AP1R	MAF and AP1 related factors	V$TCF11MAFG.01	570	590	(+)	1.000	0.823	tgaccaTGACtgagaaaccct
V$NR2F	Nuclear receptor subfamily 2 factors	V$TR2.01	584	608	(-)	0.756	0.779	aaagattagcgaaaGGTTagggttt
V$RXRF	RXR heterodimer binding sites	V$VDR_RXR.06	586	610	(-)	1.000	0.805	ttaaagattagcgaaAGGTtagggt
V$PRDF	Positive regulatory domain I binding factor	V$BLIMP1.01	586	604	(-)	1.000	0.820	attagcGAAAggttagggt
V$NBRE	NGFI-B response elements, nur subfamily of nuclear receptors	V$NBRE.01	586	600	(-)	1.000	0.874	gcgaAAGGttagggt
V$BARB	Barbiturate-inducible element box from pro+eukaryotic genes	V$BARBIE.01	587	601	(-)	1.000	0.903	agcgAAAGgttaggg
V$HOMF	Homeodomain transcription factors	V$TLX1.01	591	609	(+)	0.818	0.844	aacctttCGCTaatcttta
V$NR2F	Nuclear receptor subfamily 2 factors	V$PNR.01	595	619	(-)	0.759	0.792	cctaatagaTTAAagattagcgaaa
V$GATA	GATA binding factors	V$GATA1.02	597	609	(-)	1.000	1.000	taaaGATTagcga
V$HNF1	Hepatic Nuclear Factor 1	V$HNF1.02	598	614	(+)	1.000	0.813	cgcTAATctttaatcta
V$BRNF	Brn POU domain factors	V$BRN3.02	601	619	(+)	1.000	0.899	taatcttTAATctattagg
V$CART	Cart-1 (cartilage homeoprotein 1)	V$PHOX2.01	602	622	(-)	1.000	0.919	ctaccTAATagattaaagatt
V$CART	Cart-1 (cartilage homeoprotein 1)	V$PHOX2.01	603	623	(+)	1.000	0.898	atcttTAATctattaggtaga
V$LHXF	Lim homeodomain factors	V$ISL2.01	603	625	(+)	1.000	0.882	atctttaatctATTAggtagaaa
V$GATA	GATA binding factors	V$GATA3.02	604	616	(-)	1.000	0.928	aatAGATtaaaga
V$SATB	Special AT-rich sequence binding protein	V$SATB1.01	605	619	(-)	1.000	0.948	cctAATAgattaaag
V$CLOX	CLOX and CLOX homology (CDP) factors	V$CLOX.01	606	624	(+)	0.807	0.880	tttaATCTattaggtagaa
V$HOMF	Homeodomain transcription factors	V$HMX2.02	621	639	(+)	1.000	0.846	agaaacacAAACatttaga
V$HOMF	Homeodomain transcription factors	V$HMX2.02	626	644	(-)	0.750	0.822	taccttctAAATgtttgtg
V$DMRT	DM domain-containing transcription factors	V$DMRT1.01	642	662	(+)	0.934	0.781	gtagtttgacaccATGTctat
V$TALE	TALE homeodomain class recognizing TG motifs	V$TGIF.01	643	659	(-)	1.000	1.000	gacatggtGTCAaacta
V$SIXF	Sine oculis (SIX) homeodomain factors	V$SIX4.01	645	659	(-)	1.000	0.964	gacatgGTGTcaaac
V$FAST	FAST-1 SMAD interacting proteins	V$FAST1.01	651	667	(+)	1.000	0.895	caccatgtcTATTgagc
V$ZFIA	Zinc finger with interaction domain factors	V$ZID.01	657	669	(-)	1.000	0.858	tgGCTCaatagac
V$E2FF	E2F-myc activator/cell cycle regulator	V$E2F.02	659	675	(+)	1.000	0.849	ctattgagcCAAAcaac

V$CAAT	CCAAT binding factors	V$NFY.03	663	677	(+)	1.000	0.845	tgagCCAAacaacag
V$LHXF	Lim homeodomain factors	V$LHX6.01	669	691	(+)	1.000	0.858	aaacaacagTAATcagtttctcc
V$PDX1	Pancreatic and intestinal homeodomain transcription factor	V$IPF1.01	669	687	(+)	1.000	0.849	aaacaacagTAATcagttt
V$BRN5	Brn-5 POU domain factors	V$BRN5.04	670	692	(-)	1.000	0.886	gggagaaactgATTActgttgtt
V$HOXF	Paralog hox genes 1-8 from the four hox clusters A, B, C, D	V$HOXB7.01	670	688	(-)	0.755	0.853	gaaactGATTactgttgtt
V$MYBL	Cellular and viral myb-like transcriptional regulators	V$VMYB.01	670	682	(+)	0.817	0.882	aacAACAgtaatc
V$HBOX	Homeobox transcription factors	V$VAX2.01	671	689	(-)	1.000	0.851	agaaactgATTActgttgt
V$HBOX	Homeobox transcription factors	V$EVX1.01	672	690	(+)	0.775	0.855	caacagtaATCAgtttctc
V$PDX1	Pancreatic and intestinal homeodomain transcription factor	V$PDX1.01	674	692	(-)	0.783	0.789	gggagaaacTGATtactgt
V$RXRF	RXR heterodimer binding sites	V$VDR_RXR.03	683	707	(-)	0.765	0.750	aggtcataGGCTctagggagaaact
V$NOLF	Neuron-specific-olfactory factor	V$OLF1.02	683	705	(+)	1.000	0.884	agtttcTCCCtagagcctatgac
V$NR2F	Nuclear receptor subfamily 2 factors	V$TR4.02	689	713	(-)	1.000	0.773	ctttggAGGTcataggctctaggga
V$RXRF	RXR heterodimer binding sites	V$LXRE.02	691	715	(-)	1.000	0.694	gtctttggaGGTCataggctctagg
V$RORA	v-ERB and RAR-related orphan receptor alpha	V$RORA1.01	693	715	(-)	1.000	0.952	gtctttggaGGTCataggctcta
V$GLIF	GLI zinc finger family	V$GLI3.01	701	715	(+)	0.822	0.873	atgaCCTCcaaagac
V$DMRT	DM domain-containing transcription factors	V$DMRT1.01	710	730	(-)	0.934	0.772	gttaaaagctactATGTcttt
V$HNF1	Hepatic Nuclear Factor 1	V$HMBOX.01	722	738	(-)	1.000	0.945	tacaactgGTTAaaagc
V$GRHL	Grainyhead-like transcription factors	V$GRHL1.01	725	737	(-)	1.000	0.919	acaactGGTTaaa
V$GRHL	Grainyhead-like transcription factors	V$GRHL1.01	726	738	(+)	0.770	0.876	ttaaccAGTTgta
V$HEAT	Heat shock factors	V$HSF2.02	734	758	(+)	1.000	0.979	ttgtatagaaccAGAAgattccact
V$GREF	Glucocorticoid responsive and related elements	V$ARE.02	738	756	(-)	1.000	0.896	tggaatcttctgGTTCtat
V$ZFTR	Zinc finger transcriptional repressor	V$ZNF217.01	742	754	(-)	1.000	0.903	GAATcttctggtt
V$HEAT	Heat shock factors	V$HSF2.01	743	767	(-)	0.917	0.889	agtccgtagagtgGAATcttctggt
V$SMAD	Vertebrate SMAD family of transcription factors	V$SMAD4.01	764	772	(-)	1.000	0.995	gGTCTagtc
V$IKRS	Ikaros zinc finger family	V$IK2.01	773	785	(-)	1.000	0.993	gattGGGAattga
O$VTBP	Vertebrate TATA binding protein factor	O$VTATA.02	783	799	(+)	1.000	0.911	atcttTAAAacacactt
V$ETSF	Human and murine ETS1 factors	V$GABP.01	789	809	(-)	1.000	0.878	ctaagaagGGAAgtgtgttt

V$HOMF	Homeodomain transcription factors	V$HMX2.01	801	819	(-)	0.750	0.833	aaagcagcaCCTAagaagg
V$PRDF	Positive regulatory domain I binding factor	V$BLIMP1.01	808	826	(-)	1.000	0.815	aaaaggGAAAgcagcacct
V$ETSF	Human and murine ETS1 factors	V$SPIB.01	809	829	(-)	1.000	0.884	caaaaaagGGAAagcagcacc
V$IRFF	Interferon regulatory factors	V$IRF4.01	810	830	(-)	1.000	0.983	tcaaaaaaggGAAAgcagcac
V$KLFS	Krueppel like transcription factors	V$GKLF.01	816	832	(-)	1.000	0.900	catcaaaaaAGGGaaag
V$DICE	Downstream Immunoglobulin Control Element, critical for B cell activity and specificity	V$DICE.01	828	842	(+)	0.946	0.884	tgatGTCTacacaca
O$VTBP	Vertebrate TATA binding protein factor	O$LTATA.01	885	901	(+)	1.000	0.832	cacTATAagctaaagtc
V$PLZF	C2H2 zinc finger protein PLZF	V$PLZF.01	892	906	(+)	0.958	0.946	agcTAAAgtcccata
V$HAND	Twist subfamily of class B bHLH transcription factors	V$MESP1_2.01	896	916	(+)	1.000	0.915	aaagtccCATAtgaaagaaag
V$RUSH	SWI/SNF related nucleophosphoproteins with a RING finger DNA binding motif	V$SMARCA3.01	900	910	(+)	1.000	0.985	tcCCATAtgaa
V$BRNF	Brn POU domain factors	V$BRN2.03	912	930	(-)	1.000	0.955	aaaggatttATTTtctttc
V$ABDB	Abdominal-B type homeodomain transcription factors	V$HOXA9.01	913	929	(+)	1.000	0.873	aaagaaaataAATCctt
V$HOXC	HOX - PBX complexes	V$PBX_HOXA9.01	915	931	(-)	0.750	0.839	aaaaGGATttattttct
V$RXRF	RXR heterodimer binding sites	V$VDR_RXR.05	935	959	(-)	1.000	0.794	gagtGAAGtatccgaggctgagaga
V$DMRT	DM domain-containing transcription factors	V$DMRT2.01	954	974	(-)	0.750	0.789	cgaaaatgaTATActgagtga
V$ETSF	Human and murine ETS1 factors	V$FLI.01	961	981	(-)	0.750	0.826	gtggaaCCGAaaatgatatac
V$PRDF	Positive regulatory domain I binding factor	V$BLIMP1.01	961	979	(-)	1.000	0.814	ggaaccGAAAatgatatac
V$IRFF	Interferon regulatory factors	V$IRF3.01	963	983	(-)	1.000	0.863	gtgtggaaccGAAAatgatat
V$PAX2	PAX-2 binding sites	V$PAX2.01	967	989	(-)	0.789	0.782	gaaattgtgtggaaccgAAAAtg
V$ETSF	Human and murine ETS1 factors	V$PU1.01	967	987	(-)	1.000	0.896	aattgtgtGGAAccgaaaatg
V$STAT	Signal transducer and activator of transcription	V$STAT3.01	983	1001	(+)	0.750	0.755	caatTTCTtgcaaatctca
V$OCT1	Octamer binding protein	V$OCT1.02	987	1003	(+)	0.750	0.852	ttcTTGCaaatctcata
V$BRN5	Brn-5 POU domain factors	V$BRN5.04	990	1012	(+)	0.754	0.840	ttgcaaatctcATAAtttcacat
V$DLXF	Distal-less homeodomain transcription factors	V$DLX3.01	993	1011	(+)	1.000	0.930	caaatctcaTAATttcaca
V$HOXF	Paralog hox genes 1-8 from the four hox clusters A, B, C, D	V$HOXB8.01	994	1012	(-)	1.000	0.835	atgtgaaATTAtgagattt

V$PRDF	Positive regulatory domain I binding factor	V$PRDM1.01	996	1014	(-)	1.000	0.816	aaatgtGAAAttatgagat
V$NKXH	NKX homeodomain factors	V$NKX25.02	997	1015	(+)	1.000	0.936	tctcaTAATttcacatttt
V$PAX6	PAX-4/PAX-6 paired domain binding sites	V$PAX6.03	1012	1030	(-)	0.806	0.793	taatcACCCatcagtaaaa
V$PAX2	PAX-2 binding sites	V$PAX2.01	1013	1035	(+)	0.789	0.794	tttactgatgggtgattAAAAtc
V$RXRF	RXR heterodimer binding sites	V$VDR_RXR.04	1016	1040	(+)	0.786	0.810	actgatgGGTGattaaaatcatgca
V$LHXF	Lim homeodomain factors	V$ISL1.01	1017	1039	(-)	1.000	0.831	gcatgatttTAATcacccatcag
V$HOXF	Paralog hox genes 1-8 from the four hox clusters A, B, C, D	V$HOXC8.01	1017	1035	(-)	0.759	0.855	gattttaATCAcccatcag
V$BCDF	Bicoid-like homeodomain transcription factors	V$CRX.01	1019	1035	(-)	1.000	0.991	gatttTAATcacccatc
V$ABDB	Abdominal-B type homeodomain transcription factors	V$HOXC9.01	1020	1036	(+)	1.000	0.909	atgggtgaTTAAaatca
V$CDXF	Vertebrate caudal related homeodomain protein	V$CDX1.02	1020	1038	(-)	0.864	0.884	catgattTTAAtcacccat
V$HOXF	Paralog hox genes 1-8 from the four hox clusters A, B, C, D	V$HOXB8.01	1020	1038	(+)	1.000	0.837	atgggtgATTAaaatcatg
V$PDX1	Pancreatic and intestinal homeodomain transcription factor	V$PDX1.01	1021	1039	(-)	1.000	0.860	gcatgatttTAATcaccca
V$NKX6	NK6 homeobox transcription factors	V$NKX61.01	1021	1035	(-)	1.000	0.938	gattTTAAtcaccca
V$HOMF	Homeodomain transcription factors	V$HHEX.01	1023	1041	(-)	1.000	0.963	atgcatgatttTAATcacc
V$BRN5	Brn-5 POU domain factors	V$BRN5.02	1026	1048	(-)	0.750	0.781	aagatatatgcATGAtttaatc
V$PAX6	PAX-4/PAX-6 paired domain binding sites	V$PAX6.01	1028	1046	(-)	0.786	0.768	gatatATGCatgattttaa
V$OCT1	Octamer binding protein	V$OCT3_4.02	1028	1044	(-)	1.000	0.919	tatatGCATgattttaa
V$P53F	p53 tumor suppressor	V$P53.05	1031	1053	(-)	1.000	0.818	aatgCAAGatatatgcatgattt
V$PAX8	PAX-2/5/8 binding sites	V$PAX2.02	1031	1045	(+)	0.962	0.956	aaATCAtgcatatat
V$OCT1	Octamer binding protein	V$POU3F3.01	1033	1049	(+)	1.000	0.894	atcatGCATatatcttg
O$YTBP	Yeast TATA binding protein factor	O$SPT15.01	1034	1050	(+)	1.000	0.839	tcatgcaTATAtcttgc
V$PIT1	GHF-1 pituitary specific pou domain transcription factor	V$PIT1.01	1034	1048	(-)	0.855	0.954	aagatATATgcatga
O$YTBP	Yeast TATA binding protein factor	O$SPT15.01	1037	1053	(-)	0.759	0.831	aatgcaaGATAtatgca
V$BRN5	Brn-5 POU domain factors	V$BRN5.03	1039	1061	(-)	1.000	0.805	gTAATgaaaatgcaagatatatg
V$OCT1	Octamer binding protein	V$OCT1.02	1039	1055	(-)	1.000	0.856	aaaATGCaagatatatg

V$EVI1	EVI1-myleoid transforming protein	V$EVI1.04	1042	1058	(-)	1.000	0.746	atgaaaatgcaaGATAt
V$RXRF	RXR heterodimer binding sites	V$CAR_RXR.01	1044	1068	(-)	0.771	0.819	tgaatgGGTAatgaaaatgcaagat
V$PRDF	Positive regulatory domain I binding factor	V$BLIMP1.01	1044	1062	(-)	1.000	0.830	ggtaatGAAAatgcaagat
V$OCT1	Octamer binding protein	V$OCT3_4.01	1045	1061	(-)	0.800	0.894	gtaATGAaaatgcaaga
V$PDX1	Pancreatic and intestinal homeodomain transcription factor	V$PDX1.01	1046	1064	(+)	0.783	0.767	cttgcatttTCATtaccca
V$CDXF	Vertebrate caudal related homeodomain protein	V$CDX1.02	1047	1065	(+)	0.795	0.899	ttgcattTTCAttacccat
V$HOXF	Paralog hox genes 1-8 from the four hox clusters A, B, C, D	V$HOXA5.01	1047	1065	(-)	0.784	0.848	atgggtAATGaaatgcaa
V$HBOX	Homeobox transcription factors	V$VAX1.01	1048	1066	(-)	0.788	0.849	aatgggtAATGaaatgca
V$PARF	PAR/bZIP family	V$DBP.01	1049	1065	(-)	0.885	0.860	atgggTAATgaaaatgc
V$ABDB	Abdominal-B type homeodomain transcription factors	V$HOXA10.01	1049	1065	(-)	0.799	0.881	atgggtaaTGAAaatgc
V$HOXF	Paralog hox genes 1-8 from the four hox clusters A, B, C, D	V$HOXC8.01	1050	1068	(+)	1.000	0.861	cattttcATTAcccattca
V$SORY	SOX/SRY-sex/testis determinig and related HMG box factors	V$HBP1.01	1054	1076	(-)	1.000	0.885	ttaacagatgAATGggtaatgaa
V$HOXF	Paralog hox genes 1-8 from the four hox clusters A, B, C, D	V$NANOG.01	1055	1073	(-)	1.000	0.990	acagatgAATGggtaatga
V$CAAT	CCAAT binding factors	V$CAAT.01	1058	1072	(+)	0.856	0.916	ttacCCATtcatctg
V$HOXC	HOX - PBX complexes	V$HOX_PBX.01	1059	1075	(-)	0.833	0.860	taacAGATgaatgggta
V$HAND	Twist subfamily of class B bHLH transcription factors	V$MESP1_2.01	1059	1079	(-)	0.857	0.921	ctgttaaCAGAtgaatgggta
V$HOXH	HOX - MEIS1 heterodimers	V$MEIS1A_HOXA9.01	1061	1075	(-)	0.783	0.798	TAACagatgaatggg
V$HOMF	Homeodomain transcription factors	V$HMX3.02	1063	1081	(+)	1.000	0.939	cattcatctgTTAAcagac
V$NEUR	NeuroD, Beta2, HLH domain	V$NGN_NEUROD.01	1063	1075	(+)	1.000	0.989	cattCATCtgtta
V$HNF1	Hepatic Nuclear Factor 1	V$HNF1.04	1064	1080	(+)	1.000	0.897	attcatctGTTAacaga
V$OVOL	OVO homolog-like transcription factors	V$OVOL1.01	1066	1080	(+)	1.000	0.806	tcatctGTTAacaga
V$FKHD	Fork head domain factors	V$XFD3.01	1066	1082	(-)	1.000	0.845	tgtctgttAACAgatga
V$FKHD	Fork head domain factors	V$XFD3.01	1067	1083	(+)	1.000	0.869	catctgttAACAgacat
V$HOMF	Homeodomain transcription factors	V$HMX3.02	1068	1086	(-)	1.000	0.939	cagatgtctgTTAAcagat
V$OVOL	OVO homolog-like transcription factors	V$OVOL1.01	1069	1083	(-)	1.000	0.837	atgtctGTTAacaga

V$HNF1	Hepatic Nuclear Factor 1	V$HNF1.04	1069	1085	(-)	1.000	0.903	agatgtctGTTAacaga
V$HAND	Twist subfamily of class B bHLH transcription factors	V$TAL1BETAE47.01	1073	1093	(-)	1.000	0.872	aacagccCAGAtgtctgttaa
V$NEUR	NeuroD, Beta2, HLH domain	V$NEUROD1.01	1077	1089	(+)	1.000	0.831	cagaCATCtgggc
V$RP58	RP58 (ZFP238) zinc finger protein	V$RP58.01	1077	1089	(+)	1.000	0.909	cagaCATCtgggc
V$NEUR	NeuroD, Beta2, HLH domain	V$NEUROG.01	1078	1090	(-)	0.875	0.925	agcCCAGatgtct
V$PTF1	Pancreas transcription factor 1, heterotrimeric transcription factor	V$PTF1.01	1079	1099	(+)	0.857	0.763	gacaTCTGggctgtttctatt
V$IRFF	Interferon regulatory factors	V$IRF1.01	1085	1105	(-)	1.000	0.886	ccagaaaataGAAAcagccca
V$ZFHX	Two-handed zinc finger homeodomain transcription factors	V$AREB6.04	1086	1098	(+)	1.000	0.997	gggctGTTTctat
V$E2FF	E2F-myc activator/cell cycle regulator	V$E2F.01	1095	1111	(-)	1.000	0.775	caaaagccaGAAAatag
V$CDXF	Vertebrate caudal related homeodomain protein	V$CDX2.02	1119	1137	(-)	1.000	0.899	atctgtgtTTATggctgct
V$ABDB	Abdominal-B type homeodomain transcription factors	V$HOXB9.02	1119	1135	(+)	1.000	0.882	agcagccaTAAAcacag
V$FKHD	Fork head domain factors	V$FREAC7.01	1121	1137	(+)	1.000	0.969	cagccaTAAAcacagat
V$OCT1	Octamer binding protein	V$POU3F3.01	1130	1146	(-)	1.000	0.824	cacttGCATatctgtgt
V$GATA	GATA binding factors	V$GATA.01	1131	1143	(+)	1.000	0.944	cacaGATAtgcaa
V$OCT1	Octamer binding protein	V$OCT1.02	1135	1151	(+)	1.000	0.891	gatATGCaagtgtctct
V$PPAR	Peroxisome proliferative activated receptor homodimers	V$PPARG.01	1153	1175	(-)	0.755	0.689	ccaAAGGagactataacctacca
V$PPAR	Peroxisome proliferative activated receptor homodimers	V$PPARG.01	1153	1175	(+)	1.000	0.706	tggTAGGttatagtctcctttgg
V$NF1F	Nuclear factor 1	V$NF1.01	1169	1189	(+)	1.000	0.827	cctTTGGgtatatgcccagga
V$HMTB	Human muscle-specific Mt binding site	V$MTBF.01	1174	1182	(+)	0.808	0.901	gggtATATg
V$CTCF	CTCF and BORIS gene family, transcriptional regulators with 11 highly conserved zinc finger domains	V$CTCF.03	1176	1202	(+)	0.758	0.798	gtatatgcccaggagtGGGAaggtgaa
V$RBPF	RBPJ - kappa	V$RBPJK.02	1187	1201	(+)	1.000	0.967	ggagTGGGaaggtga
V$IRFF	Interferon regulatory factors	V$IRF7.01	1190	1210	(+)	0.821	0.870	gtggGAAGgtgaaacattaac
V$DMRT	DM domain-containing transcription factors	V$DMRT4.01	1193	1213	(+)	0.875	0.840	ggaaggtgaAACAttaaccaa
V$HNF1	Hepatic Nuclear Factor 1	V$HNF1.03	1195	1211	(-)	1.000	0.959	gGTTAatgtttcacctt

V$ZFHX	Two-handed zinc finger homeodomain transcription factors	V$AREB6.04	1197	1209	(-)	1.000	0.981	ttaatGTTTcacc
V$HOXH	HOX - MEIS1 heterodimers	V$MEIS1B_HOXA9.01	1199	1213	(+)	0.750	0.799	TGAAacattaaccaa
V$HNF1	Hepatic Nuclear Factor 1	V$HNF1.04	1202	1218	(-)	1.000	0.873	aaacattgGTTAatgtt
V$CLOX	CLOX and CLOX homology (CDP) factors	V$CDP.02	1205	1223	(+)	1.000	0.946	attaacCAATgttttacat
O$VTBP	Vertebrate TATA binding protein factor	O$MTATA.01	1209	1225	(-)	1.000	0.843	tgatgTAAAacattggt
V$CREB	cAMP-responsive element binding proteins	V$E4BP4.01	1211	1231	(-)	1.000	0.862	ttagactgatGTAAaacattg
V$E4FF	Ubiquitous GLI - Krueppel like zinc finger involved in cell cycle regulation	V$E4F.01	1214	1226	(-)	0.842	0.853	ctgATGTaaaaca
V$PARF	PAR/bZIP family	V$TEF.01	1214	1230	(-)	1.000	0.937	tagactgatGTAAaaca
V$CEBP	Ccaat/Enhancer Binding Protein	V$CEBP.02	1215	1229	(-)	0.971	0.935	agactgatGTAAaac
V$AARF	AARE binding factors	V$AARE.01	1217	1225	(+)	0.885	0.958	tTTACatca
V$FKHD	Fork head domain factors	V$FHXB.01	1229	1245	(-)	0.818	0.857	gactccAAAAcatttta
V$AP1F	AP1, Activating protein 1	V$AP1.02	1238	1248	(+)	1.000	0.874	ttgGAGTcatg
V$RORA	v-ERB and RAR-related orphan receptor alpha	V$REV-ERBA.03	1250	1272	(-)	0.750	0.796	cagcacttagGTCTctaggccca
V$BRAC	Brachyury gene, mesoderm developmental factor	V$BRACH.01	1253	1273	(-)	1.000	0.663	ccagcacttAGGTctctaggc
V$NKXH	NKX homeodomain factors	V$BAPX1.01	1259	1277	(+)	1.000	0.909	agacctAAGTgctggcaaa
V$THAP	THAP domain containing protein	V$THAP1.01	1266	1276	(+)	1.000	0.974	agtgctGGCAa
V$HEAT	Heat shock factors	V$HSF1.01	1269	1293	(-)	1.000	0.857	ttcttggctactAGAAtttgccagc
V$SORY	SOX/SRY-sex/testis determinig and related HMG box factors	V$HMGIY.01	1270	1292	(+)	1.000	0.922	ctggcaAATTctagtagccaaga
V$XBBF	X-box binding factors	V$MIF1.01	1280	1298	(-)	0.750	0.785	tctgcttcttgGCTActag
V$CAAT	CCAAT binding factors	V$NFY.03	1283	1297	(+)	1.000	0.811	gtagCCAAgaagcag
V$GREF	Glucocorticoid responsive and related elements	V$PRE.01	1294	1312	(-)	1.000	0.869	ttttcagcctcTGTTctgc
V$SORY	SOX/SRY-sex/testis determinig and related HMG box factors	V$HMGIY.01	1298	1320	(-)	1.000	0.944	gctgaaAATTttcagcctctgtt
V$E2FF	E2F-myc activator/cell cycle regulator	V$E2F1_DP1.01	1300	1316	(+)	0.782	0.811	cagaGGCTgaaaatttt
V$SORY	SOX/SRY-sex/testis determinig and related HMG box factors	V$HMGIY.01	1305	1327	(+)	1.000	0.944	gctgaaAATTttcagcaaatgag

V$STAT	Signal transducer and activator of transcription	V$STAT3.01	1308	1326	(-)	0.750	0.760	tcatTTGCtgaaaattttc
V$STAT	Signal transducer and activator of transcription	V$STAT1.01	1310	1328	(+)	0.759	0.788	aaattttcaGCAAatgaga
V$HAND	Twist subfamily of class B bHLH transcription factors	V$MESP1_2.01	1313	1333	(+)	0.893	0.915	ttttcagCAAAtgagaggtca
V$CAAT	CCAAT binding factors	V$NFY.03	1316	1330	(+)	0.750	0.829	tcagCAAAtgagagg
O$INRE	Core promoter initiator elements	O$DINR.01	1318	1328	(-)	0.969	0.968	tcTCATttgct
V$EREF	Estrogen response elements	V$ER.02	1326	1344	(+)	1.000	0.873	agagGTCAgcctgggctac
V$CP2F	CP2-erythrocyte Factor related to drosophila Elf1	V$CP2.02	1342	1360	(-)	0.875	0.842	tTCTGgaactcgctgtgta
V$XBBF	X-box binding factors	V$MIF1.01	1349	1367	(-)	0.850	0.836	atagttgttctGGAActcg
V$GREF	Glucocorticoid responsive and related elements	V$PRE.01	1355	1373	(-)	1.000	0.892	atagtgatagtTGTTctgg
V$MEF2	MEF2, myocyte-specific enhancer binding factor	V$SL1.01	1357	1379	(+)	1.000	0.841	agaacaaCTATcactataaagct
V$MYT1	MYT1 C2HC zinc finger protein	V$MYT1L.01	1357	1369	(-)	1.000	0.958	tgatAGTTgttct
O$VTBP	Vertebrate TATA binding protein factor	O$MTATA.01	1368	1384	(+)	1.000	0.939	cactaTAAAgctggacc
V$CSEN	Calsenilin, presenilin binding protein, EF hand transcription factor	V$DREAM.01	1381	1391	(-)	1.000	0.974	gaGTCAgggtc
V$ABDB	Abdominal-B type homeodomain transcription factors	V$HOXD13.01	1386	1402	(+)	1.000	0.920	tgactcgaTAAAacaac
V$GATA	GATA binding factors	V$GATA1.05	1388	1400	(+)	1.000	0.931	actcGATAaaaca
O$VTBP	Vertebrate TATA binding protein factor	O$VTATA.02	1389	1405	(+)	1.000	0.924	ctcgaTAAAacaactga
O$INRE	Core promoter initiator elements	O$DINR.01	1397	1407	(-)	1.000	0.979	gtTCAGttgtt
V$MYBL	Cellular and viral myb-like transcriptional regulators	V$CMYB.02	1397	1409	(+)	0.990	0.960	aaCAACtgaacaa
V$MYT1	MYT1 C2HC zinc finger protein	V$MYT1.02	1406	1418	(-)	1.000	0.890	tccAAGTttttgt
V$NF1F	Nuclear factor 1	V$NF1.02	1411	1431	(-)	1.000	0.873	aaagTGGCacttctccaagtt
V$NF1F	Nuclear factor 1	V$NF1.01	1411	1431	(+)	1.000	0.846	aacTTGGagaagtgccacttt
V$RUSH	SWI/SNF related nucleophosphoproteins with a RING finger DNA binding motif	V$SMARCA3.02	1417	1427	(-)	1.000	0.987	tggcACTTctc
V$HOMF	Homeodomain transcription factors	V$HMX3.01	1420	1438	(-)	1.000	0.897	gaattgaaAAGTggcactt
V$PRDF	Positive regulatory domain I binding factor	V$BLIMP1.01	1421	1439	(-)	1.000	0.833	ggaattGAAAagtggcact

V$IRFF	Interferon regulatory factors	V$IRF7.01	1422	1442	(-)	0.937	0.862	gctgGAATtgaaaagtggcac
V$ZFTR	Zinc finger transcriptional repressor	V$ZNF217.01	1434	1446	(-)	0.824	0.911	GAAGgctggaatt
V$HEAT	Heat shock factors	V$HSF1.01	1435	1459	(-)	0.857	0.852	ttgcaggaatcaTGAAggctggaat
V$ETSF	Human and murine ETS1 factors	V$ETS2.01	1442	1462	(-)	1.000	0.843	tagttgcAGGAatcatgaagg
V$STAT	Signal transducer and activator of transcription	V$STAT.01	1445	1463	(-)	1.000	0.912	atagttgcaGGAAtcatga
V$STAT	Signal transducer and activator of transcription	V$STAT1.01	1447	1465	(+)	0.759	0.795	atgattcctGCAActattt
V$BTBF	BTB/POZ (broad complex, TramTrack, Bric-a-brac/pox viruses and zinc fingers) transcription factor	V$KAISO.01	1450	1460	(+)	1.000	0.987	attcCTGCaac
V$E2FF	E2F-myc activator/cell cycle regulator	V$E2F.01	1460	1476	(-)	1.000	0.861	agtacacggGAAAatag
V$TALE	TALE homeodomain class recognizing TG motifs	V$TGIF.01	1469	1485	(+)	1.000	1.000	cgtgtactGTCAattta
V$FAST	FAST-1 SMAD interacting proteins	V$FAST1.01	1471	1487	(+)	0.850	0.821	tgtactgtcAATTtact
V$PBXC	PBX1 - MEIS1 complexes	V$PBX1_MEIS1.03	1472	1488	(-)	1.000	0.790	tagtaaatTGACagtac
V$ZF35	Zinc finger protein ZNF35	V$ZNF35.01	1484	1496	(-)	1.000	0.964	agcaggAATAgta
V$AP1R	MAF and AP1 related factors	V$MARE.01	1486	1506	(+)	1.000	0.996	ctattcctGCTGacttcgttt
V$AP1R	MAF and AP1 related factors	V$VMAF.01	1490	1510	(+)	1.000	0.845	tcctgcTGACttcgtttaaaa
V$PAX2	PAX-2 binding sites	V$PAX2.01	1501	1523	(-)	1.000	0.793	aacactggttgtttttAAACga
O$VTBP	Vertebrate TATA binding protein factor	O$MTATA.01	1501	1517	(+)	1.000	0.864	tcgttTAAAaaacaacc
V$SORY	SOX/SRY-sex/testis determinig and related HMG box factors	V$SOX9.03	1502	1524	(+)	0.760	0.820	cgtttaaaaacaaccaGTGTtg
V$CABL	C-abl DNA binding sites	V$CABL.01	1509	1519	(+)	1.000	0.976	aaAACAaccag
V$GRHL	Grainyhead-like transcription factors	V$GRHL1.01	1511	1523	(-)	1.000	0.880	aacactGGTTgtt
V$GTBX	GT box	V$ZEC.01	1513	1531	(+)	0.750	0.709	caaccagtgttGGATgcat
V$HOXC	HOX - PBX complexes	V$HOX_PBX.01	1520	1536	(+)	0.944	0.883	tgttGGATgcatgagcc
V$PAX8	PAX-2/5/8 binding sites	V$PAX2.02	1522	1536	(-)	0.923	0.936	ggCTCAtgcatccaa
V$DMRT	DM domain-containing transcription factors	V$DMRT4.01	1533	1553	(-)	0.875	0.804	aaattttttAACAttcggct
V$SORY	SOX/SRY-sex/testis determinig and related HMG box factors	V$HMGIY.01	1543	1565	(+)	1.000	0.930	ttaaaaAATTtacatattttta

V$OCT1	Octamer binding protein	V$OCT1.06	1544	1560	(-)	1.000	0.950	aatatgtaAATTtttta
V$MYT1	MYT1 C2HC zinc finger protein	V$MYT1.01	1545	1557	(+)	0.750	0.799	aaaAAATttacat
V$CREB	cAMP-responsive element binding proteins	V$E4BP4.01	1545	1565	(-)	1.000	0.854	taaaaaatatGTAAatttttt
V$FAST	FAST-1 SMAD interacting proteins	V$FAST1.02	1545	1561	(-)	0.800	0.841	aaataTGTAaattttt
V$PARF	PAR/bZIP family	V$VBP.01	1548	1564	(-)	1.000	0.886	aaaaaatatGTAAattt
V$FKHD	Fork head domain factors	V$HFH1.01	1555	1571	(-)	1.000	0.867	agaaaaTAAAaaatatg
V$CDXF	Vertebrate caudal related homeodomain protein	V$CDX2.02	1555	1573	(+)	1.000	0.864	catattttTTATtttcttt
V$EVI1	EVI1-myleoid transforming protein	V$EVI1.04	1556	1572	(-)	0.750	0.734	aagaaaataaaaAATAt
V$ABDB	Abdominal-B type homeodomain transcription factors	V$HOXD10.01	1557	1573	(-)	1.000	0.921	aaagaaaaTAAAaaata
V$STAT	Signal transducer and activator of transcription	V$STAT5.01	1562	1580	(-)	0.845	0.947	cttcTTCAaagaaaataaa
V$STAT	Signal transducer and activator of transcription	V$STAT5.01	1564	1582	(+)	1.000	0.940	tattTTCTttgaagaagat
V$LEFF	LEF1/TCF	V$LEF1.02	1565	1581	(-)	1.000	0.986	tcttcttCAAAgaaaat
V$EVI1	EVI1-myleoid transforming protein	V$EVI1.07	1570	1586	(+)	1.000	0.903	ctttgAAGAagataaaa
V$EVI1	EVI1-myleoid transforming protein	V$EVI1.06	1573	1589	(+)	1.000	0.875	tgaagaAGATaaaaaga
V$GATA	GATA binding factors	V$GATA1.03	1576	1588	(+)	1.000	0.963	agaaGATAaaaag
V$DLXF	Distal-less homeodomain transcription factors	V$DLX3.01	1590	1608	(+)	1.000	0.925	ggagatctgTAATttctaa
V$HOXF	Paralog hox genes 1-8 from the four hox clusters A, B, C, D	V$HOXB6.01	1591	1609	(-)	1.000	0.862	cttagaAATTacagatctc
V$HOXF	Paralog hox genes 1-8 from the four hox clusters A, B, C, D	V$HOXB6.01	1594	1612	(+)	1.000	0.874	atctgtAATTtctaagaaa
V$ATBF	AT-binding transcription factor	V$ATBF1.01	1597	1613	(-)	1.000	0.830	gtttcttagaAATTaca
V$STAT	Signal transducer and activator of transcription	V$STAT5.01	1597	1615	(-)	1.000	0.982	aagtTTCTtagaaattaca
V$XBBF	X-box binding factors	V$MIF1.01	1598	1616	(+)	0.850	0.768	gtaatttctaaGAAActtg
V$BCL6	POZ domain zinc finger expressed in B-Cells	V$BCL6.02	1598	1614	(-)	1.000	0.925	agtttctTAGAaattac

V$STAT	Signal transducer and activator of transcription	V$STAT5.01	1599	1617	(+)	1.000	0.971	taatTTCTaagaaacttga
V$P53F	p53 tumor suppressor	V$P53.01	1608	1630	(+)	0.844	0.743	agaaaCTTGatttggcctgctga
V$MYT1	MYT1 C2HC zinc finger protein	V$MYT1.01	1608	1620	(+)	0.750	0.757	agaAACTtgattt
V$HNF6	Onecut homeodomain factor HNF6	V$HNF6.01	1609	1625	(-)	1.000	0.827	aggccaaaTCAAgtttc
V$CP2F	CP2-erythrocyte Factor related to drosophila Elf1	V$CP2.01	1611	1629	(+)	0.909	0.902	aaCTTGatttggcctgctg
V$AP1R	MAF and AP1 related factors	V$MARE.03	1622	1642	(+)	1.000	0.840	gcctGCTGagtccagccacta
V$SP1F	GC-Box factors SP1/GC	V$SP2.01	1628	1642	(-)	1.000	0.819	tagtggctGGACtca
V$PAX2	PAX-2 binding sites	V$PAX2.01	1649	1671	(-)	1.000	0.789	gcaattttacttggctaAAACct
V$ARID	AT rich interactive domain factor	V$JARID2.01	1653	1671	(-)	1.000	0.952	gcaatTTTActtggctaaa
V$HOXH	HOX - MEIS1 heterodimers	V$MEIS1B_HOXA9.01	1659	1673	(-)	0.750	0.802	TGGCaattttacttg
V$MYT1	MYT1 C2HC zinc finger protein	V$MYT1.01	1660	1672	(-)	0.750	0.844	ggcAATTttactt
V$CLOX	CLOX and CLOX homology (CDP) factors	V$CDP.02	1666	1684	(+)	1.000	0.944	aattgcCAATtttctaaga
V$BCL6	POZ domain zinc finger expressed in B-Cells	V$BCL6.02	1672	1688	(-)	1.000	0.851	tttctctTAGAaaattg
V$PRDF	Positive regulatory domain I binding factor	V$PRDM1.01	1679	1697	(+)	1.000	0.837	ctaagaGAAAgggctagca
V$BRNF	Brn POU domain factors	V$BRN2.01	1692	1710	(-)	0.767	0.887	ctAATGagcaatgtgctag
V$CART	Cart-1 (cartilage homeoprotein 1)	V$PHOX2.01	1694	1714	(-)	1.000	0.914	tgctcTAATgagcaatgtgct
V$BRN5	Brn-5 POU domain factors	V$BRN5.04	1695	1717	(+)	1.000	0.927	gcacattgctcATTAgagcattc
V$LHXF	Lim homeodomain factors	V$LHX6.01	1695	1717	(+)	0.777	0.846	gcacattgcTCATtagagcattc
V$PDX1	Pancreatic and intestinal homeodomain transcription factor	V$IPF1.01	1695	1713	(+)	0.789	0.844	gcacattgcTCATtagagc
V$LHXF	Lim homeodomain factors	V$LHX8.01	1696	1718	(-)	0.771	0.861	agaatgctctAATGagcaatgtg
V$HOXF	Paralog hox genes 1-8 from the four hox clusters A, B, C, D	V$HOXB3.01	1696	1714	(-)	1.000	0.903	tgctcTAATgagcaatgtg
V$HBOX	Homeobox transcription factors	V$GSH2.01	1697	1715	(-)	1.000	0.969	atgctcTAATgagcaatgt

V$HBOX	Homeobox transcription factors	V$EVX1.01	1698	1716	(+)	1.000	0.876	cattgctcATTAgagcatt
V$HOXF	Paralog hox genes 1-8 from the four hox clusters A, B, C, D	V$HOXD3.01	1699	1717	(+)	0.799	0.855	attgctCATTagagcattc
V$PDX1	Pancreatic and intestinal homeodomain transcription factor	V$IPF1.01	1700	1718	(-)	1.000	0.841	agaatgctcTAATgagcaa
V$ZFTR	Zinc finger transcriptional repressor	V$ZNF217.01	1705	1717	(-)	1.000	0.903	GAATgctctaatg
V$HEAT	Heat shock factors	V$HSF2.02	1706	1730	(-)	1.000	0.960	acaggcaagctcAGAAtgctctaat
V$NFAT	Nuclear factor of activated T-cells	V$NFAT.01	1733	1751	(-)	1.000	0.960	gggtaGGAAaaaaagatt
V$CIZF	CAS interating zinc finger protein	V$NMP4.01	1736	1746	(-)	1.000	0.978	ggAAAAaaaag
V$CHOP	C/EBP homologous protein (CHOP)	V$CHOP.01	1749	1761	(+)	1.000	0.931	ccctGCAAttcc
V$ETSF	Human and murine ETS1 factors	V$CETS1P54.01	1749	1769	(-)	0.902	0.929	aacgcaCAGGaattgcaggg
V$MYBL	Cellular and viral myb-like transcriptional regulators	V$VMYB.04	1760	1772	(-)	1.000	0.903	tatAACGcacagg
V$ABDB	Abdominal-B type homeodomain transcription factors	V$HOXC9.01	1763	1779	(+)	0.884	0.831	gtgcgttaTAAAcgaaa
V$CDXF	Vertebrate caudal related homeodomain protein	V$CDX2.02	1763	1781	(-)	1.000	0.858	ggtttcgtTTATaacgcac
O$VTBP	Vertebrate TATA binding protein factor	O$VTATA.01	1766	1782	(+)	1.000	0.913	cgttaTAAAcgaaacct
V$HOMF	Homeodomain transcription factors	V$HMX2.02	1769	1787	(+)	1.000	0.837	tataaacgAAACctttcta
V$HOMF	Homeodomain transcription factors	V$HMX2.02	1774	1792	(-)	0.750	0.854	acagctagAAAGgtttcgt
V$LHXF	Lim homeodomain factors	V$ISL1.01	1784	1806	(+)	1.000	0.821	tctagctgtTAATgcaggctgtg
V$HOMF	Homeodomain transcription factors	V$BARX2.01	1786	1804	(+)	1.000	0.955	tagctgtTAATgcaggctg
V$FAST	FAST-1 SMAD interacting proteins	V$FAST1.02	1798	1814	(+)	1.000	0.900	caggcTGTGaattgaag
V$LHXF	Lim homeodomain factors	V$LHX3.02	1816	1838	(+)	1.000	0.825	aaaaaaaagcatgTAATtaatca
V$PARF	PAR/bZIP family	V$VBP.01	1819	1835	(+)	1.000	0.864	aaaaagcatGTAAttaa
V$CART	Cart-1 (cartilage homeoprotein 1)	V$S8.01	1819	1839	(-)	1.000	0.992	atgatTAATtacatgcttttt
V$LHXF	Lim homeodomain factors	V$LHX9.01	1820	1842	(+)	1.000	0.902	aaaagcatgtAATTaatcatagg
V$PDX1	Pancreatic and intestinal homeodomain transcription factor	V$IPF1.01	1820	1838	(+)	1.000	0.928	aaaagcatgTAATTtaatca

V$DLXF	Distal-less homeodomain transcription factors	V$DLX1.02	1820	1838	(+)	1.000	0.990	aaaagcatgtAATTaatca
V$LHXF	Lim homeodomain factors	V$LHX3.01	1821	1843	(-)	1.000	0.906	tcctatgaTTAAttacatgcttt
V$HOXF	Paralog hox genes 1-8 from the four hox clusters A, B, C, D	V$HOXC8.01	1821	1839	(-)	1.000	0.989	atgattaATTAcatgcttt
V$HBOX	Homeobox transcription factors	V$VAX2.01	1822	1840	(-)	1.000	0.939	tatgattaATTAcatgctt
V$BRNF	Brn POU domain factors	V$BRN4.01	1822	1840	(+)	1.000	0.912	aagcatgtaatTAATcata
V$HOMF	Homeodomain transcription factors	V$BSX.01	1822	1840	(+)	1.000	0.962	aagcatgtAATTaatcata
V$BRNF	Brn POU domain factors	V$BRN3.03	1823	1841	(-)	1.000	0.937	ctatgatTAATtacatgct
V$HBOX	Homeobox transcription factors	V$VAX1.01	1823	1841	(+)	1.000	0.938	agcatgtAATTaatcatag
V$NKX1	NK1 homeobox transcription factors	V$NKX12.01	1823	1839	(-)	1.000	0.870	atgattAATTacatgct
V$OCT1	Octamer binding protein	V$OCT1.05	1823	1839	(+)	1.000	0.954	agCATGtaattaatcat
V$ARID	AT rich interactive domain factor	V$BRIGHT.01	1823	1841	(+)	1.000	0.949	agcatgtaATTAatcatag
V$HOMF	Homeodomain transcription factors	V$BSX.01	1823	1841	(-)	1.000	0.955	ctatgattAATTacatgct
V$LHXF	Lim homeodomain factors	V$LHX3.01	1824	1846	(+)	1.000	0.850	gcatgtaaTTAAtcataggaggt
V$HOXF	Paralog hox genes 1-8 from the four hox clusters A, B, C, D	V$HOXC8.01	1824	1842	(+)	1.000	0.970	gcatgtaATTAatcatagg
V$NKX6	NK6 homeobox transcription factors	V$NKX63.01	1824	1838	(+)	1.000	0.879	gcatgTAATtaatca
V$OCT1	Octamer binding protein	V$OCT1.03	1824	1840	(-)	1.000	0.939	tatgattaATTAcatgc
V$PAXH	PAX homeodomain binding sites	V$PAX6_HD.01	1824	1838	(-)	1.000	0.934	tgattAATTacatgc
V$ABDB	Abdominal-B type homeodomain transcription factors	V$HOXA9.01	1824	1840	(+)	1.000	0.937	gcatgtaattAATCata
V$NKX1	NK1 homeobox transcription factors	V$NKX12.01	1824	1840	(+)	1.000	0.882	gcatgtAATTaatcata
V$CART	Cart-1 (cartilage homeoprotein 1)	V$S8.01	1824	1844	(+)	1.000	0.997	gcatgTAATtaatcataggag
V$HOXF	Paralog hox genes 1-8 from the four hox clusters A, B, C, D	V$HOXD8.01	1825	1843	(-)	1.000	0.816	tcctatgATTAattacatg
V$PAXH	PAX homeodomain binding sites	V$PAX4.02	1825	1839	(+)	1.000	0.912	catgtAATTaatcat

V$PDX1	Pancreatic and intestinal homeodomain transcription factor	V$IPF1.01	1825	1843	(-)	1.000	0.927	tcctatgatTAATtacatg
V$LHXF	Lim homeodomain factors	V$LMX1B.01	1825	1847	(-)	1.000	0.941	aacctcctatgatTAATtacatg
V$NKX6	NK6 homeobox transcription factors	V$NKX61.01	1825	1839	(-)	1.000	1.000	atgaTTAAttacatg
V$DLXF	Distal-less homeodomain transcription factors	V$DLX2.01	1825	1843	(-)	1.000	0.973	tcctatgattAATTacatg
V$HOXC	HOX - PBX complexes	V$PBX_HOXA9.01	1826	1842	(-)	1.000	0.849	cctaTGATtaattacat
V$PIT1	GHF-1 pituitary specific pou domain transcription factor	V$PIT1.02	1826	1840	(-)	1.000	0.904	tatgaTTAAttacat
V$BRNF	Brn POU domain factors	V$BRN3.03	1826	1844	(+)	1.000	0.929	atgtaatTAATcataggag
V$MEF2	MEF2, myocyte-specific enhancer binding factor	V$SL1.01	1826	1848	(-)	1.000	0.872	caacctcCTATgattaattacat
V$ATBF	AT-binding transcription factor	V$ATBF1.01	1827	1843	(-)	1.000	0.808	tcctatgattAATTaca
V$PIT1	GHF-1 pituitary specific pou domain transcription factor	V$PIT1.02	1827	1841	(+)	1.000	0.907	tgtaaTTAAtcatag
V$NKX6	NK6 homeobox transcription factors	V$NKX63.01	1828	1842	(+)	1.000	0.894	gtaatTAATcatagg
V$BCDF	Bicoid-like homeodomain transcription factors	V$CRX.01	1828	1844	(+)	1.000	0.941	gtaatTAATcataggag
V$ZBPF	Zinc binding protein factors	V$ZNF219.01	1837	1859	(-)	1.000	0.939	gcgaacaCCCCcaacctcctatg
V$KLFS	Krueppel like transcription factors	V$KLF6.01	1844	1860	(+)	1.000	0.920	ggttGGGGgtgttcgct
V$GLIF	GLI zinc finger family	V$ZIC2.01	1845	1859	(-)	1.000	0.933	gcgaacaCCCCcaac
V$MYBL	Cellular and viral myb-like transcriptional regulators	V$VMYB.02	1862	1874	(-)	0.820	0.901	tgtAACTgaagct
V$PAX6	PAX-4/PAX-6 paired domain binding sites	V$PAX6.02	1863	1881	(+)	0.865	0.899	gcttcagttACAGgggaga
V$INSM	Insulinoma associated factors	V$INSM1.01	1870	1882	(+)	1.000	0.901	ttacaGGGGagaa
V$ZNFP	Zinc finger proteins	V$SZF1.01	1872	1896	(+)	0.801	0.859	acaGGGGagaagctggacaaggcac
V$MOKF	Mouse Krueppel like factor	V$MOK2.02	1886	1906	(-)	1.000	0.988	ctaggtcctagtgCCTTgtcc
V$MYT1	MYT1 C2HC zinc finger protein	V$MYT1L.01	1907	1919	(-)	1.000	0.945	ggatAGTTgcctt

V$NF1F	Nuclear factor 1	V$NF1.03	1912	1932	(+)	0.853	0.923	aactatccaccctGGCAggaa
V$KLFS	Krueppel like transcription factors	V$EKLF.01	1914	1930	(-)	1.000	0.926	cctgccaGGGTggatag
V$ETSF	Human and murine ETS1 factors	V$ETS2.01	1921	1941	(+)	1.000	0.911	ccctggcAGGAatttcttgct
V$BTBF	BTB/POZ (broad complex, TramTrack, Bric-a-brac/pox viruses and zinc fingers) transcription factor	V$KAISO.01	1923	1933	(-)	1.000	0.994	attcCTGCcag
V$SORY	SOX/SRY-sex/testis determinig and related HMG box factors	V$SOX2.01	1948	1970	(+)	1.000	0.992	tcagacaACAAaggcatagagag
V$LEFF	LEF1/TCF	V$LEF1.01	1949	1965	(+)	1.000	0.927	cagacaaCAAAggcata
V$CAAT	CCAAT binding factors	V$NFY.04	1965	1979	(-)	1.000	0.920	aaaaCCAAtctctct
V$HNF6	Onecut homeodomain factor HNF6	V$OC2.01	1967	1983	(-)	0.750	0.823	aaagaaAACCaatctct
V$CAAT	CCAAT binding factors	V$NFY.03	1996	2010	(+)	1.000	0.825	ccacCCAAccagcag
