# Supplementary figures and images for: A short upstream promoter region mediates transcriptional regulation of the mouse doublecortin gene in differentiating neurons
Source: BMC Neurosci. 2010 May 28;11:64. doi: 10.1186/1471-2202-11-64 (PMC2891791; doi:10.1186/1471-2202-11-64)

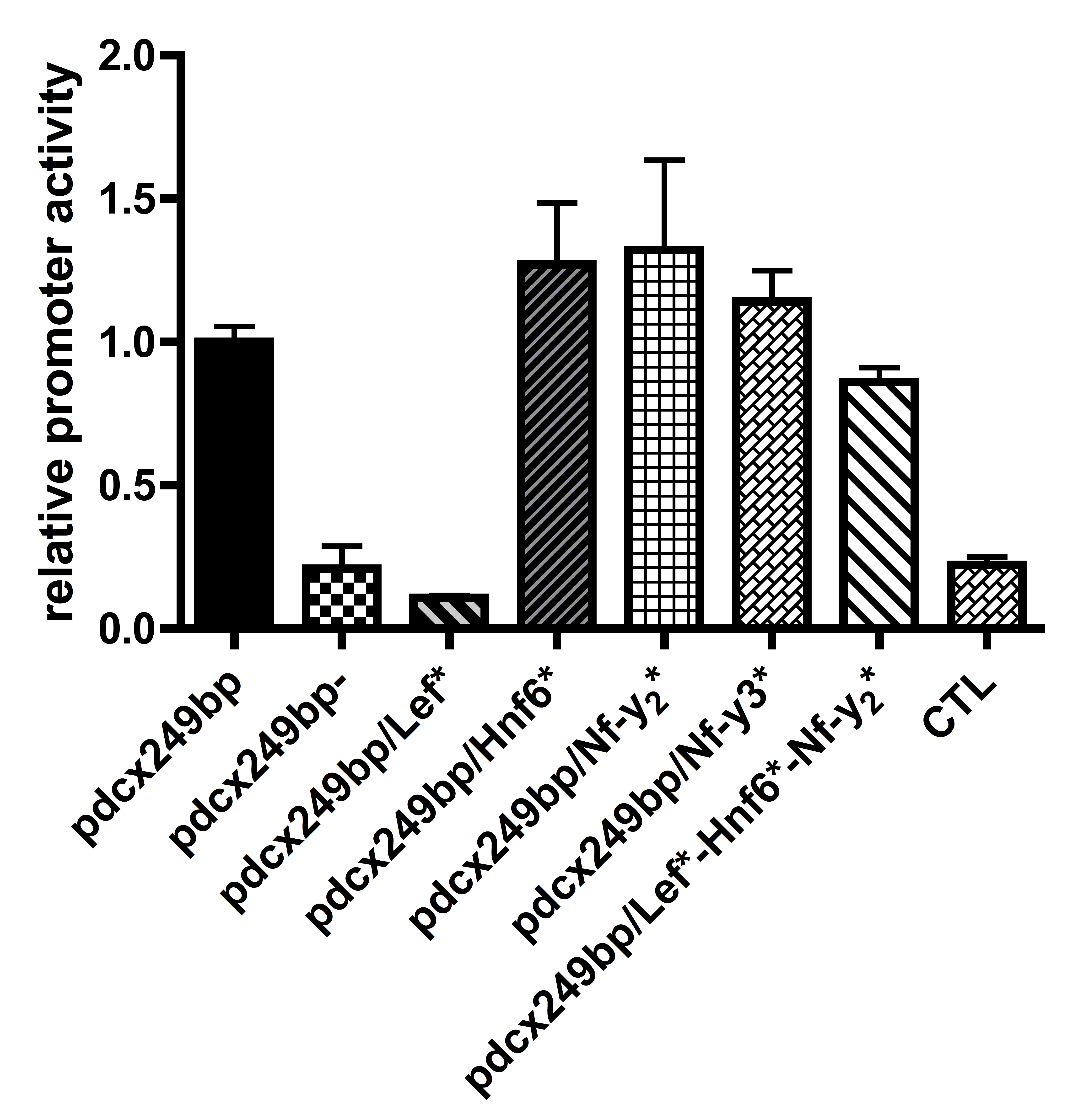

Supplement: Additional file 2 — Simultaneous mutation of the LEF, HNF6 and NF-Y binding sites in Dcx promoter. ESR1 cells were transfected with the indicated constructs at day of differentiation 0 (DD0) and luciferase activity relative to β-galactosidase activity was determined 48 hours later. The different constructs were pdcx249bp-Luc, 79bp-deleted pdcx249bp-Luc construct (pdcx249bp--Luc) and pdcx249bp-Luc with mutated binding site (pdcx249bp/Lef*, pdcx249bp/Hnf6*, pdcx249bp/Nf-y2*, pdcx249bp/Nf-y3* and pdcx249bp/Lef*/Hnf6*/Nf-y2*) and the promoterless control (CTL). The activity of each construct is expressed relative to that of the pdcx249bp-Luc construct, set arbitrarily to 1. Each value represents the mean ± SD of one transfection experiment, performed in triplicate. Asterisks mean significantly different from pdcx249bp values at P < 0.05 (*) or P < 0.01 (**). [file 1471-2202-11-64-S2.TIFF]
